# Emerging field of few-layered intercalated 2D materials†

**DOI:** 10.1039/d0na00987c

**Published:** 2021-01-15

**Authors:** Qing Cao, Fabian Grote, Marleen Huβmann, Siegfried Eigler

**Affiliations:** Institute of Chemistry and Biochemistry, Freie Universität Berlin Takustraβe 3 14195 Berlin Germany siegfried.eigler@fu-berlin.de

## Abstract

The chemistry and physics of intercalated layered 2D materials (2DMs) are the focus of this review article. Special attention is given to intercalated bilayer and few-layer systems. Thereby, intercalated few-layers of graphene and transition metal dichalcogenides play the major role; however, also other intercalated 2DMs develop fascinating properties with thinning down. Here, we briefly introduce the historical background of intercalation and explain concepts, which become relevent with intercalating few-layers. Then, we describe various synthetic methods to yield intercalated 2DMs and focus next on current research directions, which are superconductivity, band gap tuning, magnetism, optical properties, energy storage and chemical reactions. We focus on major breakthroughs in all introduced sections and give an outlook to this emerging field of research.

## Abstract 2

The chemistry and physics of intercalated layered 2D materials (2DMs) are the focus of this review article.

## Graphical abstract

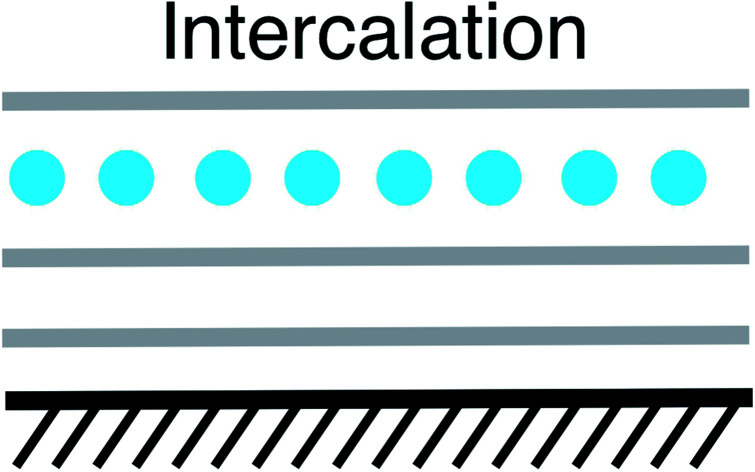

## 1 Introduction

Our mobile and rechargeable world is based on intercalation chemistry. Thus, it is not surprising that the Nobel Prize in Chemistry 2019 was given to the pioneers of the Li-ion battery, Goodenough, Whittingham, and Yoshino.^1^ The pioneering work of Whittingham was the report on the reversible intercalation of layered TiS_2_ by Li-ions. The work of Goodenough led to the discovery of layered cobalt oxide as cathode material and the work of Yoshino explored graphite as anode material. Now, energy can be stored by collecting photons from the sun or collecting kinetic energy from wind or water and used at will.

With the discovery of graphene by Geim and Novoselov, which was awarded with a Nobel Prize in 2010 it was revealed that properties of materials change with thinning down to the single layer.^2^ Thus, novel materials emerged and were studied by physicists. In addition, the chemistry of 2D materials (2DMs) came into the focus of research and new functionalization principles were developed, both of covalent and non-covalent nature.^3–5^ Surface physics and surface chemistry, both accelerated in recent years and thus, researchers realized that 2DMs properties can be tailored by surface modification, such as the deposition of metal particles or organic donor and acceptor type molecules, respectively.^6,7^ In general, countless investigations are being made into the chemistry and physics of 2DMs and a practically infinite variety is emerging. Thereby, transition metal dichalcogenides (TMDCs) or 2D allotropes of Si, Bi, P and others possess different band structures. Thus, 2DMs are insulators, topological insulators, semiconductors, conductors, semimetals or semiconductors with direct or indirect bandgap.^8^ In recent years, research on the fabrication of heterostructures is targeted by artificially assembling those 2DMs with combining and extending physical properties.^9^

Historically, the term intercalation and intercalation chemistry was coined by Rüdorff and Hoffmann in 1938,^10^ although intercalation in the form of swelling was already observed in 1841 by Schafhaeutl.^11^ Key-results of graphite intercalation compounds (GICs) are summarized and explained by M. S. Dresselhaus and G. Dresselhaus.^12^ Thereby, intercalation is performed in bulk materials and various GICs are described, which are divided into acceptor and donor type GICs, respectively. Examples are graphite sulfate and potassium graphite, which are the best studied materials so far.^12^

However, in light of recent developments, it is more obvious than ever that the intercalation of stacks of 2DMs is of particular interest. While a single layer of a 2DM cannot be intercalated, bilayers (2Ls) and few-layers can already be intercalated, as summarized in this review. As known from the experience of graphene physics compared to graphite physics, the properties of single- and few-layered 2DMs differ from the bulk *e.g.* due to confinement of charge carriers.^13^ Because of the ease of language, the expression “intercalation of 2DMs” is used in this article for the intercalation of any few-layered 2DMs. In addition, we propose to expand the terminology of intercalation, which should not be limited to the traditional process in which atoms or ions penetrate the galleries of layered compounds. In this review we use the term “intercalated” to describe the final product, which can be regarded as an intercalated layered system. Accordingly, a 2DM may be intercalated between molecular layers of *e.g.* organic molecules. In this example surface science and intercalation mix. A single layer of a 2DM may be intercalated between other 2DMs, such as graphene encapsulated between hBN layers, *e.g.* by artificial assembly, a structure which can also be termed as van der Waals (vdW) structure.^14^ With this expanded view of intercalation, a huge research area can be identified and summarized.

**Fig. 1:**
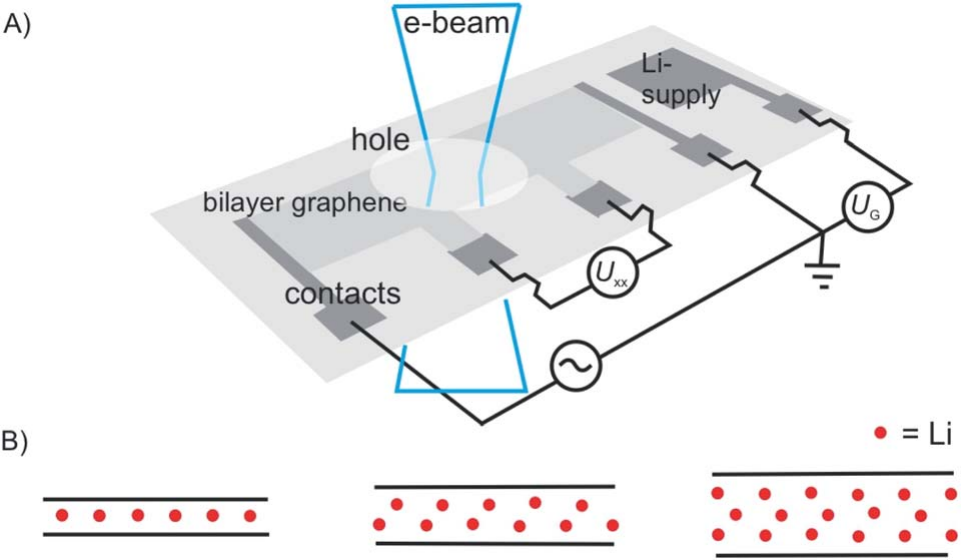
Illustration of *in situ* transmission electron microscopy of the Li-intercalation process in suspended 2L graphene, triggered in a device. (A) Illustration of the device on a Si_3_N_4_-covered Si substrate. (B) from left to right: side-views of expected ‘conventional’ C_6_LiC_6_ configuration and of the experiment matching structures with 2L Li crystal and 3L Li crystal.^15^

As a motivating and fascinating recent example, we want to highlight the intercalation of 2L graphene by Li, a process which is related to the anode process of a Li-ion battery.^15^ A 2L of graphene was mounted on a grid in the SALVE microscope (sub-Angstrom-low-voltage-electron microscope) and the intercalation was triggered by a 5 V potential (Fig. 1A). As depicted in Fig. 1, the process of intercalation can be followed microscopically with atomic resolution. The growth of the Li crystal between 2L graphene was conducted and filtering the structure of the 2L graphene led to the conclusion that the Li crystal differs from the expected packing for C_6_LiC_6_ (Fig. 1B), but can be explained by the formation of a 2L and a trilayer (3L) of Li. Thus, those results differ from bulk lithium graphite with the formular LiC_6_, since more Li atoms can be intercalated between two layers compared to the bulk.

Other review articles and books are available, however summarizing primarily the intercalation of bulk materials. In particular the review by Stark *et al.* summarizes intercalation processes not only on bulk, but also 2D level.^12,16–20^ Thus, here we keep history and bulk information short, since it is covered in the above mentioned articles. Further, we introduce important principles relevant for few-layer, trilayer (3L), bilayer (2L) and monolayer (1L) intercalation. Moreover, we highlight the recent advances. More specifically, we will focus on concepts, the synthetic access to intercalated 2DMs and introduce common methods, such as vapor transport, solvent based or electrochemical methods and artificial assembly. Next, we highlight recent results and properties of intercalated 2DM systems.

## 2 Concept of staging

Here, we introduce concepts and definitions relevant for 2DMs intercalation, which are derived from definitions introduced for GICs. We note that the historical background and intercalation concepts for bulk materials are summarized in the excellent perspective article of Lerf.^20^ In particular, staging and charge transfer are of importance to describe the structure and properties of intercalation compounds. With thinning down the bulk to the 1L additional considerations become important, which are bridging intercalation and surface science.

### 2.1 Staging in bulk materials

**Fig. 2:**
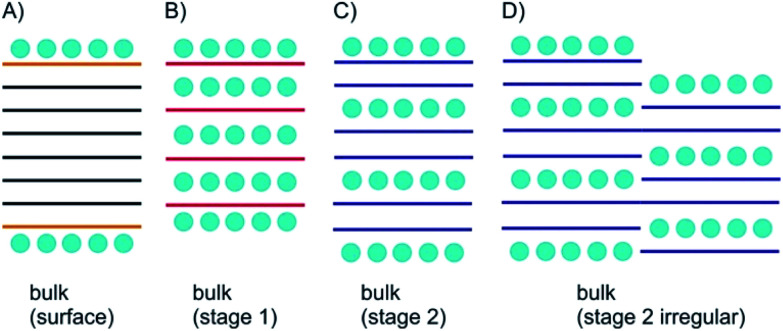
Concept of bulk surface functionalization/intercalation. (A) Layered bulk material with surface functionalization; the interior is not influenced; (B) stage 1 intercalation compound; (C) stage 2 intercalation compound; (D) irregular, ill-defined stage 2 intercalation compound.

As depicted in Fig. 2A surface functionalization differs from intercalation. The most important concept for describing intercalated materials is staging, which is used to describe the structure of layered materials with guest atoms, ions or molecules. This concept is best studied for GICs. Accordingly, stage *n* was defined with *n* as number of layers. Thus, if we define A as materials layer and B as intercalant layer, stage 1 possesses an ABABAB sequence (Fig. 2B), stage 2 AABAABAAB sequence (Fig. 2C), stage 3 AAABAAAB sequence *etc.*

However, also mixed systems are possible with *e.g.* partially-filled layers, in particular occurring for natural systems, as a consequence of the kinetic control of intercalation (Fig. 2D). It is obvious that a small intercalant must enter the galleries of a layered bulk material from the side and intercalation starts from all rims at the same time. However, for the example of a stage 2 compound the hypothetical left rim intercalant does not know at which layer the intercalant on the right rim starts. Such a mismatch cannot be corrected and will consequently lead to a mixed layer sequence. For a 2L material there is only one gallery to be intercalated. With the given examples, the difference between surface manipulation, by interacting molecules or atoms, and intercalation becomes clear, since with intercalation layers, the bulk can be influenced from inside. With thinning the layered materials, the surface becomes more important for manipulating materials properties and thus, interesting effects emerge as outlined in the next chapters.

### 2.2 From bulk to few-layers of intercalated 2D materials

**Fig. 3:**
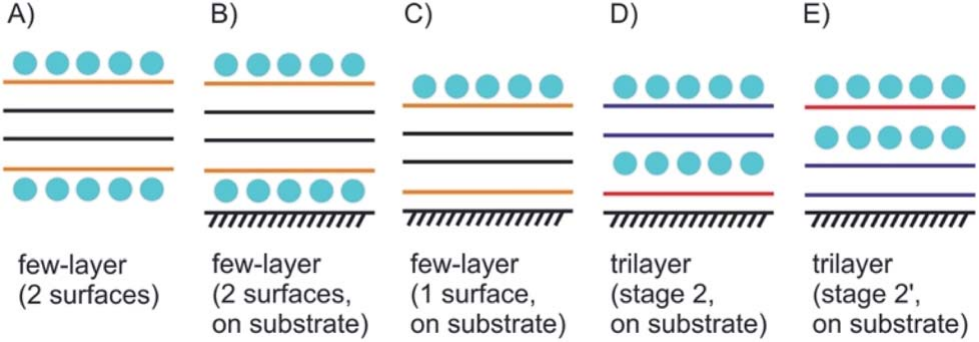
Concept of few-layer surface functionalization/intercalation. (A) Surface functionalized few-layer material, here the interior 2L differ from the outer layers; (B) as in (A), but with substrate influence leading to Janus-type properties; (C) few-layered material with intercalation only on the top; (D) and (E) illustration of stage 2 and stage 2′ intercalation for 3L materials.

**Fig. 4:**
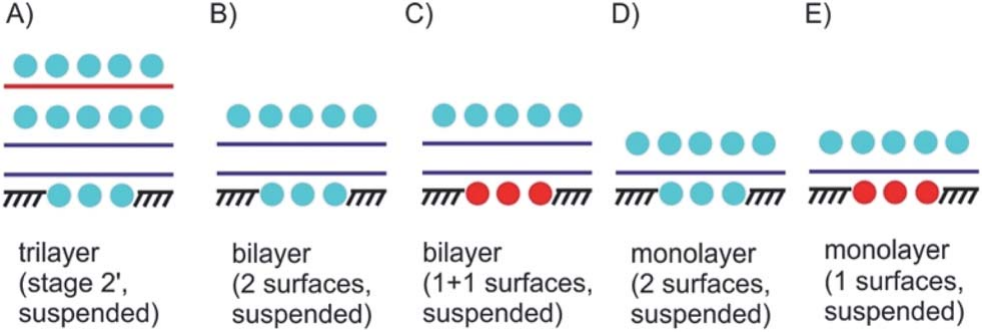
Suspended 3L, 2L and 1L materials with surface functionalization/intercalation. (A) 3L stage 2′ intercalated and bottom surface functionalization; (B) surface functionalized suspended 2L; (C) Janus-type surface functionalized 2L; (D) 1L material intercalated between molecular layers; (E) Janus-type suspended 1L material; (D) and (E) could be considered as 2D 1L intercalated between molecular layers.

Few-layered 2DMs are obtained at first sight with thinning down from bulk.^21^ However, the concept of staging loses its validity with reaching 1L, instead surface science describes the systems. Here, we introduce some of the possible structures for few-layered systems, followed by three, two and one layers of 2DMs (Fig. 3 and 4).

As shown in Fig. 3A the four layers (4L) are an example of a few-layered material with the surface influenced by molecules or atoms. In that example with 4L the orange marked layers can be distinguished from the inner black marked layers and there is consequently a junction between orange and black layers. However, for more than roughly six layers surface functionalization does not play a crucial role for the interior layers, since the proportion of surface layers to the interior layers becomes minor.

Even for the example of 4Ls the substrate plays an important role. As illustrated in Fig. 3B and C, with considering a substrate, Janus-type functionalization (different functionalities on two sides) is realized no matter whether atoms or molecules are placed between the substrate and the 2DM or not. For the 3L examples in Fig. 3D and E the concept of staging is adopted, here, with stage 2 as an example. Starting with a 3L on a substrate and an intercalant preferring stage 2 formation, two different configurations can be considered, and thus, either a 1L or 2L is on the substrate. Here, we introduce stage 2 and stage 2′, respectively, to differentiate between those two cases. The example in Fig. 3D starts from the top like a stage 2 compound. Thus, we assign stage 2′ to the example in Fig. 3E.

In Fig. 4A a stage 2′ structure is shown, however, in contrast to Fig. 3E the structure is suspended, which can be realized by placing the structure on top of a hole in the substrate. A similar structure is shown in Fig. 1A for a 2L of graphene. In general, with suspending few-layered 2DMs transmission is possible, however, also surface manipulation, such as doping becomes possible accounting for different surfaces. This is the point where surface science plays the decisive role in manipulating 2DMs. For a 2L both surfaces can be accessed by molecules or atoms, which are the same (Fig. 4B) or different ones (Fig. 4C); the latter leading to Janus-type functionalization. Taking the concept of staging into account a stage 2 configuration can be realized for a 2L, although no intercalation occurs. For a 1L similar surface functionalization is possible, as shown in Fig. 4D and E. In those examples, the 1L is however intercalated between molecular layers and thus, surface science and intercalation chemistry merge.

**Fig. 5:**
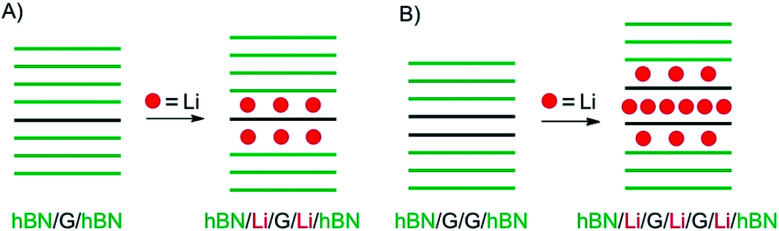
Study of device-based electrochemical intercalation of Li in heterostructures of (A) graphene (G) artificially intercalated in hBN and (B) 2L graphene artificially intercalated in hBN.

In a recent study, intercalation of vdW heterostructures of graphene and 2L graphene, respectively, was studied in an electrochemical device (as outlined in chapter 3).^22^ The structures prepared are illustrated in Fig. 5A and B. As shown in Fig. 5A intercalation proceeds at the interface between hBN and graphene, on both sides. However, the properties differ from pure surface functionalization or a stage 1 bulk intercalation compound, due to the interaction of Li and hBN (about 30L on each side). The authors conclude that Li is randomly distributed, and the amount of Li atoms is significantly lower compared to the bulk with carrier densities of about 7 × 10^13^ cm^−2^, corresponding to a stoichiometry of LiC_60_.

In contrast, a 2L of graphene intercalated in hBN, as shown in Fig. 5B, behaves differently because intercalation of the 2L allows a much higher loading compared to the graphene/hBN interface. Here, the carrier density was determined to 5.5 × 10^14^ cm^−2^, with a contribution of 4.6 × 10^14^ cm^−2^ for the graphene/graphene interface.

### 2.3 Considerations on geometry and charge carrier densities

When approaching a 1L of a 2DM, or by stacking few-layers of 2DMs on top of each other or by intercalating materials, atomic-scale illustrations become important to imagine the dimensions. In this regard, structures are often evaluated by transport measurements in devices. As a result, the mobility of charge carriers is determined, either in field effect transistors or in magnetic fields by Hall-bar measurements. Finally, taking the dimensions of the device into account a charge carrier density is derived with the unit cm^−2^ for 2DMs. However, for an atomic imagination the unit cell dimensions are important to take into account. For graphene, the unit cell contains two carbon atoms, with two equal lattice vectors. The calculated area for one carbon atom is 0.026195 nm^2^ or 0.026195 × 10^−14^ cm^2^ (order of magnitude comparable to typical charge carrier densities), respectively.^23^ This means that there are 38 × 10^14^ C-atoms per cm^2^. For the example illustrated in Fig. 5, this consideration means that there is one charge on about 14 C-atoms, taking the 2L structure and interaction of all intercalated Li-atoms into account.

For MoS_2_ the area of the unit cell is 0.088 nm^2^ including one Mo and two S atoms, of which one points up and one down. Accordingly, there are 11 × 10^14^ Mo-atoms per cm^2^ and 22 × 10^14^ S-atoms per cm^2^. In another example the intercalated heterostructure MoS_2_/Li/graphene was analyzed by density functional theory calculations,^24^ with a charge carrier density of 3.6 × 10^14^ cm^−2^ for graphene and 6.0 × 10^14^ cm^−2^ for MoS_2_. According to the considerations above those value relate to one charge carrier on about 10.6 C-atoms and 3.7 S-atoms, respectively.

## 3 Synthetic access to intercalated 2D materials

### 3.1 Intercalation by vapor-transport

**Fig. 6:**
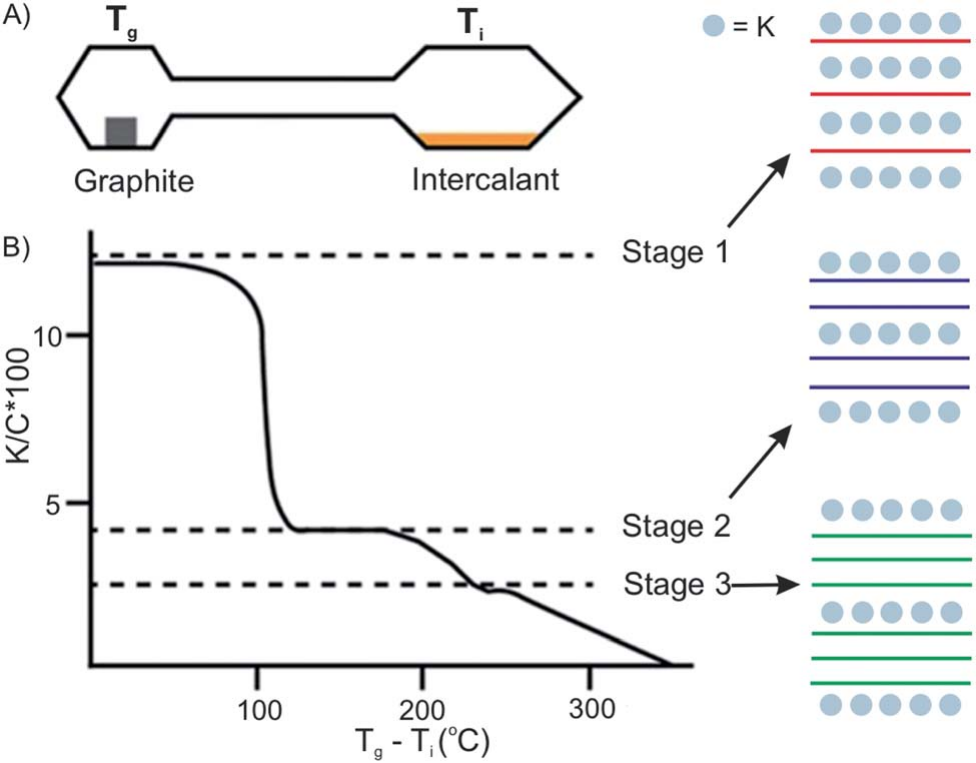
Two-zone thermal transport for potassium into graphite and stage control by temperature difference. Reproduced from ref. 16 with permission from Wiley-VCH Verlag GmbH & Co, Copyright 2019.

The vapor transport method usually proceeds under vacuum or an inert gas atmosphere in a sealed two-zone cell (Fig. 6A).^12^ The intercalants and host materials are placed in separate zones, which are heated under different or same temperatures, respectively. The success of intercalation depends on controlling the temperature of the host zone and intercalant zone. Also, the stage of the intercalation compounds can be controlled by temperature adjustments. The temperature of the two zones can be different or isotherm, depending on the chemical and physical properties of the intercalant and the host material.^25^ For example, the stage of the prototype intercalation compound K-GICs is controlled by the temperature difference *T*_g_ − *T*_i_ (*T*_g_: temperature of graphite; *T*_i_: temperature of intercalant) as shown in Fig. 6B.^26^ The higher stages are formed at a smaller *T*_g_ − *T*_i_.

In the case of halogen intercalation, typical preparation temperatures are around 20 °C to 60 °C for *T*_g_. Owing to the high threshold vapor pressure of the halogens, such as Br_2_,^27^ IBr and ICl, *T*_i_ is set from −30 °C to 60 °C to control the pressure in the reaction cell.^28^ Other parameters, including heating time, the size of host materials, the amount of intercalant, and the volume and shape of ampoule also affect the homogeneity and in general the quality of the final compound.^12,29,30^

**Fig. 7:**
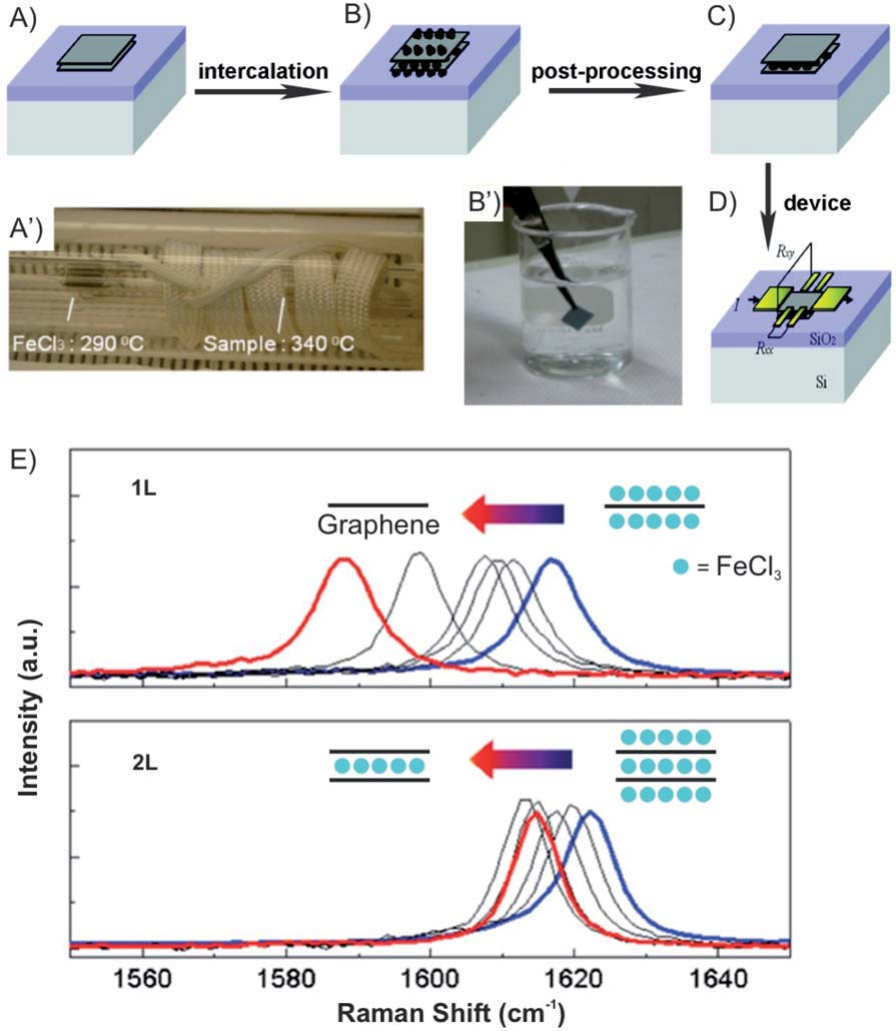
Fabrication of an FeCl_3_ intercalated 2L graphene device. (A) Pristine and (B) after FeCl_3_ intercalation of 2L graphene deposited on Si/SiO_2_ substrate. (A′) The sealed tube is placed in the furnace. (B′) Sufficient washing with acetone (C) removes the adsorbed molecules from the surface. (D) Deposition of electrodes using standard lithography fabrication techniques. (E) Gradual downshifts of the Raman G peaks is observed (after intercalation without air exposure; blue) and after washing in acetone for 0, 0.16, 0.5, 1 (black), and 12 h (red). Adapted from ref. 47 with permission from the American Chemical Society, Copyright 2011.

Vapor transport intercalation is the most widely applied method for the intercalation of bulk materials, because it works for most of the possible hosts, such as graphite,^12^ fullerenes,^31^ single-walled carbon nanotubes,^32^ hBN^33^ and TMDCs (MoS_2_, TaS_2_, WS_2_ and WSe_2_)^34–36^ and a variety of intercalants, such as alkali metal (K, Rb and Cs)^26,37,38^ or their alloys,^39,40^ halogens (Br_2_)^27^ or interhalogens (IBr and ICl),^28,41^ metal halides (FeCl_3_, AlCl_3_, SbCl_5_ and AsF_5_)^42–45^ and organic molecules (pyridine, cyclopropylamine).^46^ This method is already well developed for bulk intercalation. Thus, it can be easily and directly applied to 2D host materials using the same equipment (two-zone cell) and similar experimental conditions (temperature). The 2D host is normally deposited on a substrate or a manufactured device that can be directly inserted into the reaction cell, as depicted in Fig. 7A–D, illustrating FeCl_3_ intercalation.^47^ With reduced lateral dimensions, the intercalation rate of few-layered materials is much faster compared to the bulk. The synthesis of stage 1 alkali metal (K, Rb) GICs usually takes more than 12 h,^48^ while only 5 min are needed for the intercalation of 1–15L of graphene.^49^

**Fig. 8:**
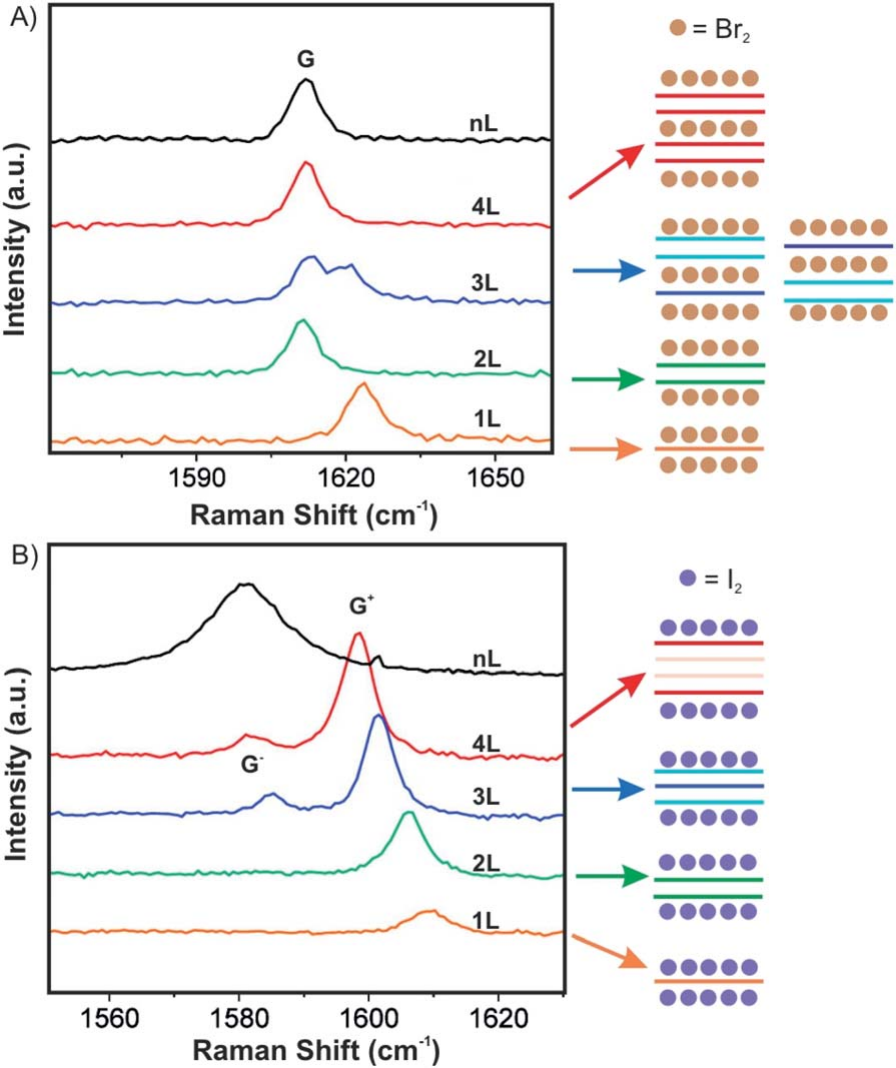
Raman spectra of few layer graphene materials exposed to (A) Br_2_ and (B) I_2_ and the derived intercalation models. Adapted from ref. 50 with permission from the American Chemical Society, Copyright 2009.

Over the past decade, progress has been made in few-layered 2DMs intercalation by the vapor transport method. The 2DM intercalation compounds were studied and some showed similarities to their bulk intercalation compound, such as the formed stage,^49,50^ which offers good references for the investigation and characterization of few-layered 2DMs intercalation. For few-layered 2DMs the effect of surface adsorption of intercalants becomes non-negligible as discussed in chapter 2. For example, Br_2_ and I_2_ have been attempted to intercalate in mechanically exfoliated 1–4L of graphene on a p-type Si/SiO_2_ wafer.^50,51^ The wafer with host materials was placed in one zone of a glass cell, while liquid halogen was placed in another zone at a temperature of 10 °C to keep a constant vapor pressure in the cell. To avoid any impurities, the tube cell was initially evacuated to 2.7 × 10^−5^ mbar and the liquid halogen was frozen and thawed several times to remove dissolved gases. After 1 h of Br_2_ exposure, Br_2_ was successfully intercalated into 3L and 4L of graphene, respectively. In contrast, for 2L and 1L graphene, Br_2_ was symmetrically adsorbed on the top and bottom surfaces. Those results are consistent with the observations made for the Br_2_-GIC, with stage 2 as the lowest reported stage.^27^ Accordingly, the model as shown in Fig. 8A is concluded from Raman spectra of the intercalation compounds. A single G peak near 1612 cm^−1^ is found for 2L and 4L graphene, which are symmetrically intercalated and doped, similar to the bulk Br_2_-GIC reference. However, the 1L showed an upshift of the G peak to 1614 cm^−1^, which relates to a higher charge carrier concentration for the single graphene layer. For intercalated 3L graphene, two G peaks are detected, which indicates two different types of inequivalent graphene layers as a result of asymmetric intercalation.

Few-layer graphene was also exposed to I_2_. Raman spectra for 3L and more layers treated with I_2_ showed two G peaks (Fig. 8B). This means I_2_ adsorbs only on the top and the bottom of few-layer graphene without intercalation, possibly because the longer I_2_ bond length does not allow an intercalation structure.^52^ Similar to bulk intercalation, Br_2_ is also the only diatomic nonpolar halogen molecule that can be easily intercalated into graphite. However, iodine in interhalogen compounds such as ICl and IBr can be intercalated.^53^

**Fig. 9:**
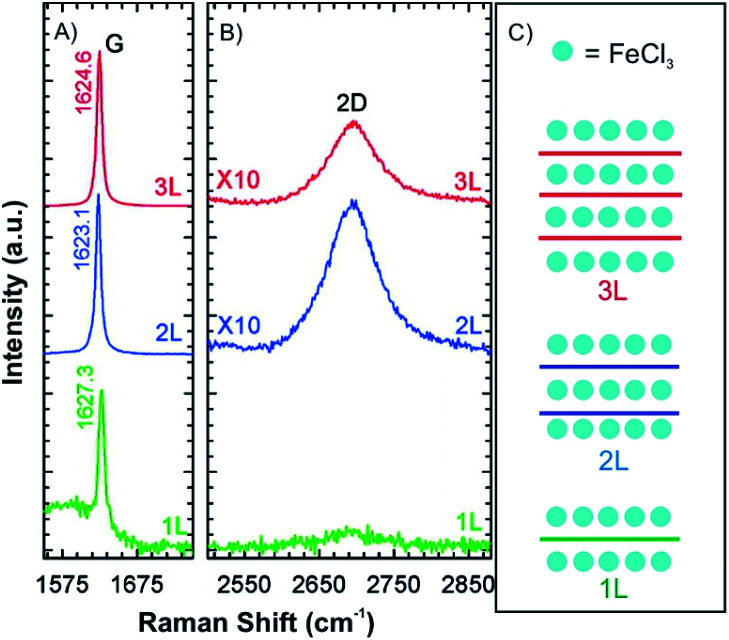
(A) G peak, (B) 2D peak and (C) schematic illustrations of stage 1 for 1L, 2L and 3L graphene–FeCl_3_ intercalation compounds. Adapted from ref. 56 with permission from the American Chemical Society, Copyright 2011.

Compared to alkali metal intercalation compounds, it is reported that FeCl_3_ intercalation compounds are relatively stable at ambient conditions.^54^ Those materials can be safely removed from their encapsulating ampoules for short periods of time, and therefore, provide prototype materials for the measurements to determine properties and further exploratory investigations. Due to that, FeCl_3_ intercalation was investigated on the 2D level.^47,55–58^ Accordingly, 1-4L graphene flakes were intercalated by FeCl_3_ to a stage 1 intercalation compound (FeCl_3_-few-layered graphene (FLGs), Fig. 9).^56^ Owing to the highly hygroscopic property of FeCl_3_, it was heated at 393 K for more than 90 min to remove any residual water. Next, FeCl_3_ and the host were sealed in an ampoule and inserted in an oven at a reaction temperature of 613 K for 6 h. The Raman spectra for 1L, 2L and 3L graphene display an upshift of the G peak and a change of the 2D peak from a multi- to a single-peak structure. This is an indication of electronic decoupling of the layers by the intercalant for 2L and 3L graphene intercalated by FeCl_3_. The single G peak also indicates uniform adsorption and intercalation of layers by FeCl_3_ (Fig. 9A). For 1L graphene both surfaces adsorb FeCl_3_ and the highest upshift of the G peak is observed at 1627 cm^−1^, compared to 1623 cm^−1^ for FeCl_3_ intercalated 2L and FeCl_3_ intercalated 3L, respectively. However, multiple G peaks also have been reported in the Raman spectra of stage 1 FeCl_3_-FLGs.^56–58^ There are two explanations for this observation: (1) FeCl_3_ molecules only intercalate between interlayers without adsorbing on the surface layers of graphene. The lower G_1_ peak is a signature of a graphene sheet with only one adjacent FeCl_3_ layer, whereas the higher shift G_2_ peak characterizes a graphene sheet sandwiched between two FeCl_3_ layers.^58^ (2) The additional G peak is caused by nonuniform intercalation, due to desorption of FeCl_3_ during cooling down in vacuum. To prove the hypotheses, FeCl_3_-FLGs were exposed to air by two ways. In the first approach, FeCl_3_-FLGs were immediately removed from the hot glass tube. In the second approach, the glass tube was first air-cooled to room temperature while still sealed and then the samples were removed. The samples obtained from the first method exhibited a single G peak and a higher doping level, which was stable up to one month in air.^56^ Moreover, as shown in Fig. 7B and C, adsorbed FeCl_3_ on graphene surface can be simply removed by washing in acetone. According to the Raman spectrum, the G peak shifts down continuously with increased washing time. Finally, a 1L is recovered with a normal G peak position, which decreased to ∼1580 cm^−1^, while the G peak of 2L graphene did not shift back to the undoped value (Fig. 7E). This difference indicates that the intercalated molecules are more stable owing to the protection between graphene layers, but would be eventually deintercalated by more extensive rinsing in acetone or other solvents such as hydrochloric acid.^47^

Through vapor transport intercalation, a lot of intercalant and host pairs have been fabricated on the bulk level (as mentioned above), but only few are reported for few-layered 2DMs. As the vapor transport method can directly be applied for few-layered 2DMs intercalation, more intercalants and host materials can be investigated in the future. However, the instability of the intercalation compounds hampers the real-time characterization and thus, also limits further investigations.

### 3.2 Solvent-based intercalation

Nonelectrochemical solvent-based intercalation is easy to apply, compared to other methods, since no special equipment or reaction conditions are necessary. Typically, the host material is soaked in a solution of the intercalant. The intercalation process is often accompanied by chemical reactions, enabling intercalation or post reactions. The most representative example is the synthesis of graphite oxide by Hummers method.^59^ In the first step, graphite is dispersed in sulfuric acid with an oxidant to form graphite sulfate, an intercalation compound with the stoichiometry [C_24_^+^HSO_4_^−^·2H_2_SO_4_]_*n*_.^10,60^ During this process the oxidant p-dopes graphite enabling intercalation of hydrogensulfate counterions and sulfuric acid molecules.^61^ With adding more oxidant, such as potassium permanganate further chemical modifications occur on both sides of the interlayers and finally covalent C–O bonds are formed. In this way, graphite oxide is formed, which can be delaminated to graphene oxide.^3^

**Fig. 10:**
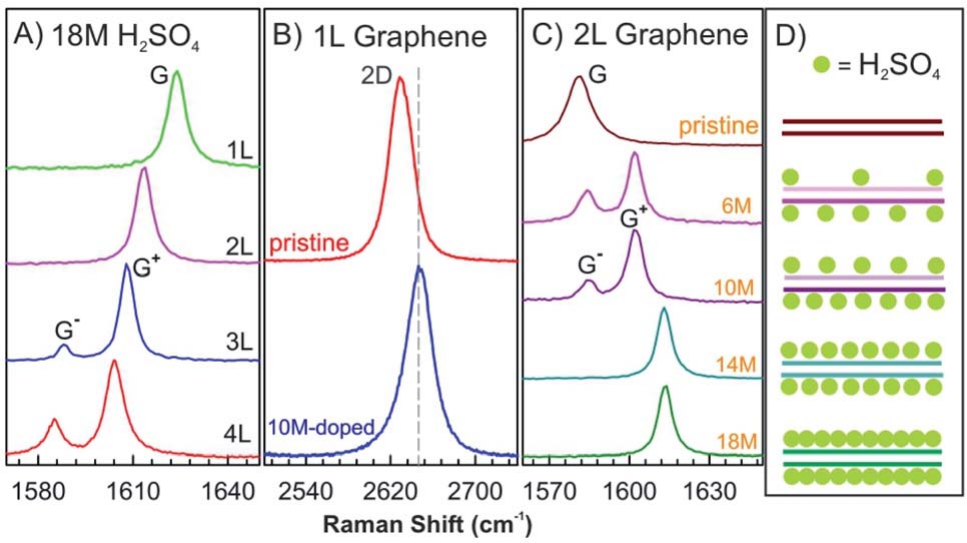
(A) *In situ* Raman spectra of the G peak of 1–4L graphene samples dipped into 18 M sulfuric acid. (B) The 2D peak of pristine 1L graphene and that doped by 10 M sulfuric acids. (C) *In situ* Raman spectra of the G peak of the 2L graphene doped by different sulfuric acid concentrations of 18 M, 14 M, 10 M, and 6 M and a pristine BLG as a reference. (D) Structural model of 2L graphene structures doped with sulfuric acid derived from Raman spectra in (C). Adapted from ref. 62 with permission from the American Physical Society, Copyright 2010.

Nevertheless, in the absence of oxidizing agents, this reaction works differently for 1–4L of graphene hosts, for which sulfuric acid molecules are found to be only physically adsorbed on the top and bottom layers without intercalation.^62^ The G peaks of 1–4L of graphene in 18 M sulfuric acid show similar Raman spectra as observed for graphene few-layers with adsorbed I_2_, although the chemical doping of I_2_ vapours on graphene is quite different (Fig. 8B and 10A). The 2D peak of 1L graphene upshifts by about 10 cm^−1^ after dipping into 10 M sulfuric acid, as shown in Fig. 10B, which is a typical characteristic of hole doping in graphene layers.^63^ As shown in Fig. 10C and D, the doping level of the top and bottom graphene layers can be asymmetric by adjusting the concentration of sulfuric acid to 6 M and 10 M, respectively. At a low concentration of sulfuric acid, the molecules are not completely adsorbed on the two surface layers, which causes the splitting of the G peak, indicating two graphene layers of different doping degree.

**Fig. 11:**
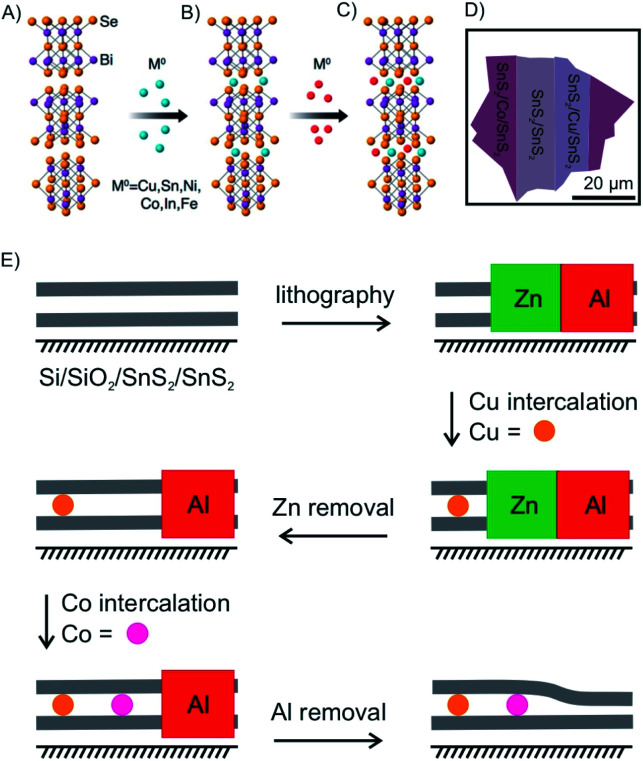
Intercalation of (A and B) single and multiple (C) zero-valent metal atoms into Bi_2_Se_3_. Adapted from ref. 66 with permission from the American Chemical Society, Copyright 2015. (D) Schematic illustration of seamlessly integrated n-type SnS_2_, p-type Cu–SnS_2_ and metallic Co–SnS_2_ within a single nanosheet. (E) Schematics of the spatially controlled intercalation process for 2L SnS_2_.^67^

Another advantage of the solvent-based intercalation is that zero-valent metal intercalation in 2D host materials can be realized, which is difficult to achieve with other intercalation methods. As illustrated in Fig. 11A a variety of zero-valent metal atoms, including Ag, Au, Co, Cu, Fe, In, Ni, and Sn, were intercalated into 2D layered Bi_2_Se_3_ nanoribbons (50 nm thick).^64^ The zerovalent guest species were generated by a disproportionation redox reaction in solution or by carbonyl decomposition (Table 1) in a refluxing solution, and then intercalated into the layered Bi_2_Se_3_ structure. The atom% of intercalant is controlled by either the concentration or the reaction time. Among all the metal atoms, Cu showed the highest intercalation concentration up to 60 atom%, which is much larger than concentrations of compounds synthesized by heating or electrochemical insertion (below ∼3 atom%).^65^ The Cu-intercalated nanoribbons appeared reddish, close to the colour of Cu metal, suggesting high concentrations of zero-valent copper. Additionally, 30 different ordered multiple pairs of zero-valent atomic species (Cu, Sn, Ni, Co, In and Fe) were also intercalated into Bi_2_Se_3_ nanoribbons by a stepwise combination of different solvent-based intercalation processes to form 2D alloys inside the host lattice (Fig. 11A and B). Depending on the type of intercalant and their intercalating order, the intercalant exhibits a variety of ordered and disordered structures (including superlattices and charge density waves). In most cases, the intercalant remains at zero valence.^66^ Furthermore, chemical vapor deposition (CVD) grown n-type semiconducting 2L SnS_2_, which was intercalated with Cu and Co atoms, is transformed into a p-type semiconductor and a highly conductive metal.^67^ Combining this method with lithography, spatially controlled intercalation can be realized to seamlessly integrate n-type and p-type semiconductors and metals in 2DMs, which is difficult to achieve with mechanical transfer or other methods (Fig. 11D and E).^67^ These methods have been further extended to intercalate various TMDCs (MoS_2_, MoSe_2_, NbSe_2_, WS_2_, Sb_2_Te_3_, In_2_Se_3_, GaSe)^68,69^ and non-TMDs, such as MoO_3_,^70^ as well as incorporating dual metal elements into those materials. As apparent from the reported results it seems that the solvent-based method to intercalate zero-valent metals in 2DMs is universal, although more investigations are necessary.

**Table 1:** Summary of reactions to generate zerovalent species, and precursor chemistry. Adapted with permission from ref. 64 from the American Chemical Society, Copyright 2012^a^

Intercalant	Precursor(s)	Reaction
Copper	Tetrakis (acetonitrile) copper(i) hexafluorophosphate	2Cu_(aq)_^+^ → Cu_(aq)_^2+^ + Cu(0)
Silver	Silver nitrate; 0.1 g 5,5,7,12,12,14-hexamethyl-1,4,8,11-tetraazocyclotetradecane	2Ag^+^ + L → AgL^2+^ + Ag(0)
Tin	Stannous chloride; 0.1 g tartaric acid	2Sn^2+^ → Sn^4+^ + Sn(0)
Gold	Gold(i) chloride or chlorotristriphenylphosphine gold(i)	3Au_(aq)_^+^ → Au_(aq)_^3+^ + 2Au(0)
Indium	Indium(i) chloride	3InCl ↔ InCl_3_ + 2In(0)
Cobalt	Dicobalt octacarbonyl	Co_2_(CO)_8_ → 8CO + 2Co(0)
Iron	Iron pentacarbonyl	Fe(CO)_5_ → 5CO + Fe (0)
Nickel	Nickel(ii) nitrate pentahydrate; hydrazine hydrate	2Ni^2+^ + N_2_H_4_ + 4OH^−^ → 2Ni(0) + N_2_ + 4H_2_O

a ^1^L = tetraazocyclic amine ligand.

Solution-phase intercalation has also been applied to MXenes,^71–73^ a new family of 2D layered materials discovered in 2011.^74^ Organic molecules^72,73^ and alkali metal ions^71,75^ have been investigated for the intercalation of solid MXene nanosheets. For example, hydrazine monohydrate, hydrazine monohydrate dissolved in *N*,*N*-dimethylformamide (DMF), urea and dimethyl sulfoxide (DMSO) were successfully intercalated into hydrofluoric acid (HF) modified 2D MXene f-Ti_3_C_2_ (Ti_3_C_2_(OH)_*x*_O_*y*_F_*z*_).^72^ To prove the universality of this method rather than the exclusive property of f-Ti_3_C_2_, other MXenes Ti_3_CN and TiNbC were also intercalated by hydrazine monohydrate. The intercalation process can be reversed by heating the intercalated material above the boiling point of the intercalated species leading to a recovery of the c-lattice parameter. DMSO-intercalated f-Ti_3_C_2_ can be exfoliated due to its hygroscopic character by sonication in water affording a stable colloidal solution of separate sheets, from which a Ti_3_C_2_ film can be prepared on an Al membrane. Although there are many studies on bulk MXenes intercalation,^75–78^ investigations conducted on few-layered MXenes remains a field of research which is in progress.

Solvent-based intercalation can be applied easily to few-layered 2DMs by immersing the substrate with the 2D host adsorbed on the surface into the solution of the intercalant.^62,79^ Unlike electrochemical intercalation, solvent-based intercalation can intercalate insulating host materials, such as hBN.^80^ The concentration of intercalants is adjustable by the concentration of solutions, however precise control is hard to achieve. It is reported that these intercalation compounds are more stable than those formed by the vapor-transport method, and can thus be easily characterized.^71,75^ In addition, the intercalation may lead to exfoliation of the layered material or is reversible. These properties can be exploited either for preparing few-layered 2DMs, 2D intercalated materials or exploited in applications.

### 3.3 Electrochemical intercalation

**Fig. 12:**
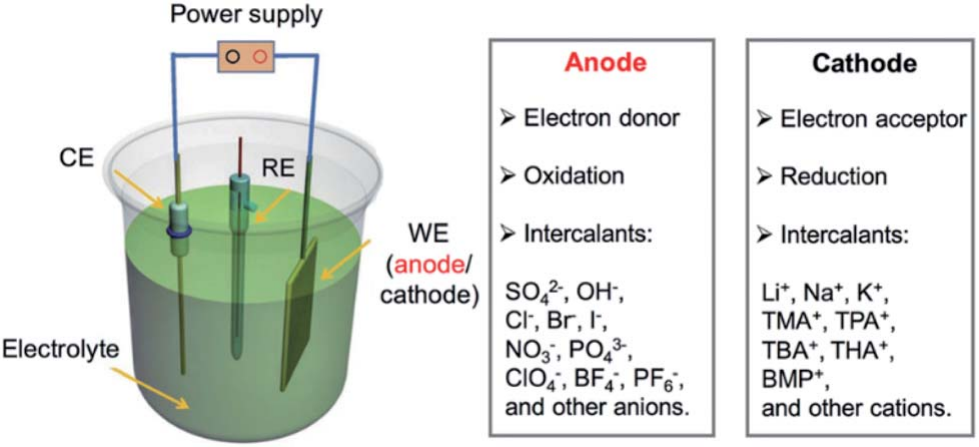
Configuration of an electrochemical cell for bulk intercalation and typical reactions as well as typical intercalants. Reproduced from ref. 81 with permission from Wiley-VCH Verlag GmbH & Co, Copyright 2020.

Electrochemical intercalation allows to reversibly intercalate cations or anions into a layered host material driven by an external bias. For bulk intercalation, this is typically carried out in a three-electrode setup as depicted in Fig. 12. The host material serves as the working electrode (WE); therefore, it must be electrically conductive. The counter electrode (CE) is typically made of an inert metal plate or wire, *e.g.* Pt. A reference electrode (RE) provides a known potential (*e.g.* Ag/AgCl or calomel) that is used to reference the relative potentials of the WE and CE during the experiment. The electrodes are placed in an aqueous, non-aqueous or solid-state electrolyte containing a salt for ion conductivity and the intercalant.

A direct or alternating current between the WE and CE is applied by an external power supply. The applied voltage is measured *versus* the potential between the RE and the WE. The intercalant enters the galleries of the host either *via* the vdW gaps or through defects of the top layer.^82^ The intercalation process can be followed *in situ* by the response of the current to the applied voltage. By further increasing the voltage, the electrochemical intercalation can be used to functionalize or exfoliate few-layered 2DMs from a bulk sample. In aqueous solutions, *e.g.* GICs can be further oxidized to graphite oxide, which is subsequently delaminated to yield graphene oxide.^83^ This approach is not limited to graphite but can be applied to a wide variety of 2DMs such as black phosphorous, TMDCs and transition metal carbides, as recently reviewed elsewhere.^81^

Moving from the bulk to few-layered materials, the size of the experimental setup decreases accordingly. Microfabrication techniques are therefore required to fabricate electrochemical devices from few-layered 2DMs. The electrodes must be connected to a voltage source meter, either by placing on a conductive substrate or by contacting the flakes directly from the top by metal electrodes deposited onto the 2DM. In both cases the sample can lose electrical contact during the experiment due to swelling of the sample or deformation of the electrodes. The disadvantageous effect of swelling on the electrode contact may depend on the 2DMs thickness and should therefore be the smallest for a 2L device.

**Fig. 13:**
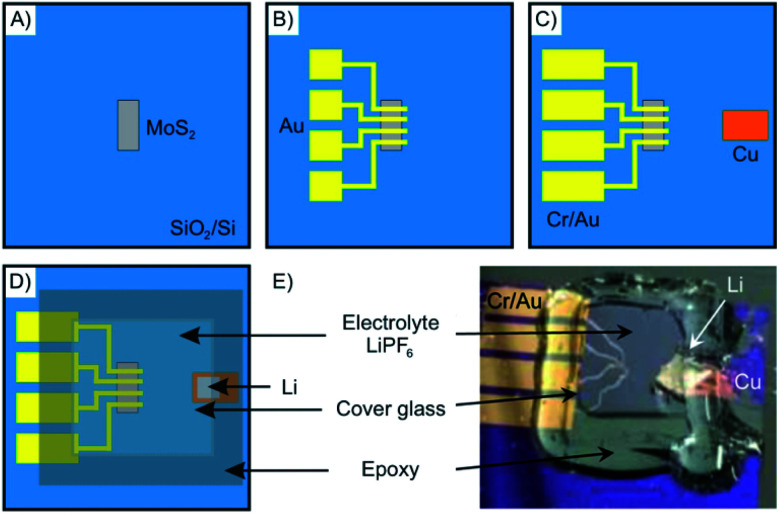
Schematics showing the fabrication steps of an electrochemical device for *in situ* monitoring of Li intercalation into nanosheets of MoS_2_. (A) Mechanically exfoliated MoS_2_ flakes are deposited onto a Si/SiO_2_-wafer. (B and C) Metal electrodes are deposited *via* e-beam lithography and shadow mask evaporation. (D) The electrolyte is applied on top of the electrodes and a cover glass is used to sandwich the device that is sealed by epoxy resin to avoid oxidation. (E) Photograph of the as-prepared electrochemical intercalation device. Adapted from ref. 84 with permission from the American Chemical Society, Copyright 2015.

As an example, in Fig. 13 the fabrication of a microscopic electrochemical intercalation device is depicted. The device enabled investigating the process of intercalation of Li into 2–50 nm thin MoS_2_. Such a typical device is fabricated starting by mechanical exfoliation of the 2DM. It is then dry-transferred onto a suitable substrate such as a Si/SiO_2_ wafer (Fig. 13A). The 2DM can be subsequently etched into any desired shape to allow for example the *in situ* measurement of the Hall effect. The metal electrodes are then deposited by electron-beam lithography and physical vapor deposition (Fig. 13B and C). In the last fabrication step, the electrolyte solution containing the intercalant is applied onto the device (Fig. 13D and E). Measuring the small currents of the micrometer sized devices is challenging, however, possible.

In the case of few-layer intercalation of black phosphorous, the measured currents are in the range of tens of nano-ampere.^85^ Zhao *et al.* encapsulated single and few-layers of graphene between hBN layers for shielding from the environment and to avoid parasitic currents originating from reactions in the electrolyte.^22^ The gold contacts used to contact the graphene layer were sealed using SU-8 resist to suppress any corrosion reactions at higher voltages.

The electrochemical intercalation between vdW heterostructures of different 2DMs is a huge new field of intercalation research. Zhao *et al.* not only used hBN to encapsule graphene layers and to avoid side reactions, but also studied the intercalation of Li ions at the interface of a single layer of graphene covered on both sides by hBN.^22^ Other vdW heterostructures of graphene and MoS_2_ were studied by Oakes *et al.* showing a large influence of strain at the interface of the two materials due to the lattice mismatch on the intercalation process.^86^ In another study Bediako *et al.* investigated Li intercalation at the interface of heterostructures of hBN, graphene, MoS_2_ and MoSe_2_.^87^ This growing new field of research is also reviewed elsewhere.^88^

**Fig. 14:**
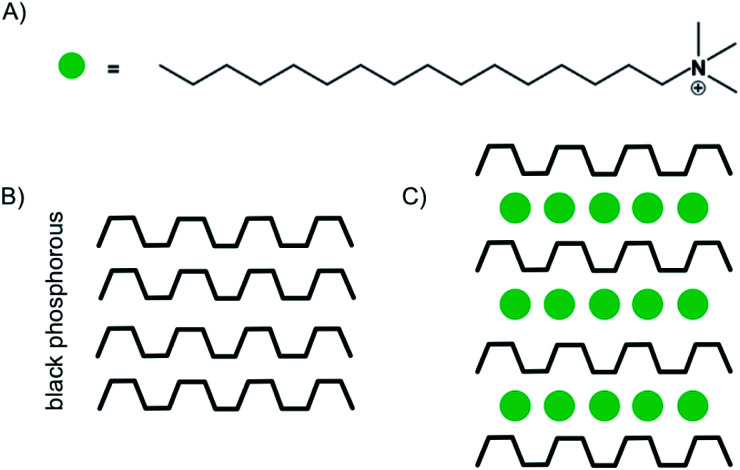
(A) Illustration of the chemical structure of the cetyltrimethylammonium cation. (B) Schematic illustration of black phosphorous. (C) Schematic illustration of the stage 1 intercalation compound.^85^

Despite the possibility to electrochemical intercalation of a wide range of both anions, such as sulfates,^89^ nitrates^90^ and hexafluorophosphates^91^ and cations into bulk materials, most of the recent reports on the intercalation of few-layered materials are focused on the intercalation by alkali metals, while examples of anion intercalation are scarce.^89^ The intercalation of Li is by far the most studied process, which is motivated by the impact of its large-scale use in Li-ion battery technology. The intercalation of Li into few-layered 2DMs may help to better understand the underlying processes and guide further improvements of the technology.^84,92,93^ As shown in the introductory example (Fig. 1), Kühne *et al.* showed superdense ordering of Li in 2L graphene.^15^ They conclude that a 2L of graphene may spread more easily upon intercalation compared to its bulk counterpart, showcasing the differences between few-layer and bulk materials. In another study, they further showed very fast Li diffusion into 2L graphene with a diffusion coefficient as high as 7 × 10^−5^ cm^2^ s^−1^.^93^ Aside from energy storage applications, electrochemical intercalation of charged organic molecules is used to fabricate well defined organic/vdW-hybrid heterostructures.^85,94^ By applying a potential of −3 V, Wang *et al.* intercalated multi-layered black phosphorous nanosheets with cetyltrimethylammonium cations (Fig. 14A). The intercalation could be followed by the response of the electrochemical gate current measured *versus* the applied voltage. The interlayer distance increased from 5.24 Å to 11.21 Å as illustrated in Fig. 14B and C. Thereby, superlattices of alternating molecule and 2DM layers were formed in which the individual phosphorene layers are electronically decoupled from each other. The decoupling of the phosphorene layers leads to an increased optical bandgap, as observed by *in situ* photoluminescence measurements.^85^

Recently, He *et al.* performed the electrochemical intercalation of the neutral organic semiconductor molecule perylene-3,4,9,10-tetracarboxylic dianhydride (PTCDA) into few-layered MoS_2_ demonstrating a powerful design scheme for the fabrication of well-defined organic devices.^95^ This may bridge the gap from the research of 2DMs and vdW heterostructures and organic semiconductor research. Electrochemical intercalation of 2DMs enables a high control over the intercalation process by controlling the applied external bias. However, the 2DM must be conductive, excluding insulating materials such as hBN. Moreover, electrochemical intercalation requires the fabrication of microscale devices which increases the techniques complexity.

### 3.4 Artificial assembly

Artificial assembly, as sketched in the introduction, provides the opportunity to access distinct heterostructures to previously introduced conventional intercalation methods. The structures to be fabricated are not limited to certain ions or small molecules, which tend to intercalate specific hosts. Thus, the artificial assembly serves chances to integrate novel building blocks for the fabrication of few-layered intercalation compounds. However, the increasing freedom of parameters leads to complexity and thus a process, which is less controllable. For example, the integrity of the lattice of 1Ls may be harmed during exfoliation and transfer. Thereby cracks or folds may be formed. Furthermore, each process may introduce contaminants. Moreover, controlling the respective orientations in stacked lattices, twisted layers can be formed in which however unique properties emerge from lattice mismatch.^96–98^ Furthermore, the orientation of ions/molecules or the number of layers are other degrees of freedom to be considered. However, with overcoming those issues, extraordinary structures with outstanding properties are created, as outlined in chapter 4.

The mechanical transfer of 2DMs is based on overcoming vdW forces of stacked layers. It is a method to design structures of twisted layers,^97–99^ containing molecules,^100,101^ or of various 2DMs, such as graphene, hBN, TMDCs, black phosphorous or silicene. The yielded structures are also termed as heterostructures or vdW heterostructures.^102–104^

**Fig. 15:**
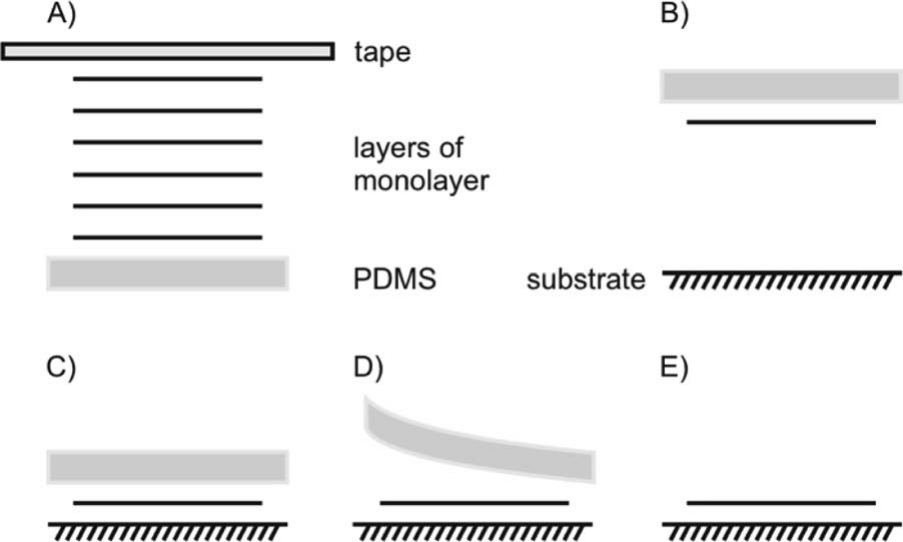
Dry transfer with viscoelastic stamp of PDMS. (A) Tape exfoliation of bulk 2DM, (B) exfoliated 1L on PDMS, upside down attached to a glass slide, that PDMS and the target substrate can be aligned by a micromanipulator, (C) contacting PDMS/1L and substrate, (D) lift off of PDMS leaving the 1L on the substrate due to their stronger interaction, (E) transferred 1L on new substrate.

The most common transfer method for flakes is a dry stamp technique using polydimethylsiloxane (PDMS) as illustrated in Fig. 15. It is an easily handled, clean, fast and reliable technique compared to others, like vdW pick up, usage of a sacrificial layer or wedging method. Those methods have been extensively compared by Frisenda *et al.*^105^

With a PDMS stamp a 1L of bulk 2DMs can also be delaminated, a process, which is similar to the first invented scotch tape method (Fig. 15A). For the transfer, the flake is picked-up by the PDMS stamp, which is attached to a glass slide. The glass slide is fixed to a micromanipulator. Due to the transparency of PDMS, the alignment to an underlying flake or substrate can be traced under an optical microscope (Fig. 15B). After bringing PDMS and substrate in contact, PDMS can be slowly peeled off leaving the flake behind on the substrate (Fig. 15C–E).

**Fig. 16:**
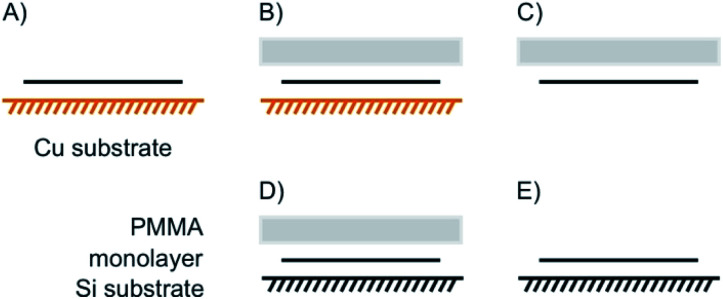
PMMA supported etching transfer commonly used for large areas of CVD grown materials. (A) 1L on CVD supporting substrate (*e.g*. copper for CVD graphene). (B) Spin casting of PMMA on top of the 1L. (C) Removal of the substrate by etching. (D) Wet or dry transfer onto new substrate; wet transfer: after etching, PMMA/1L is washed in a water bath. By decreasing the water level, the 2DM can be transferred on a new substrate, which is placed at the bottom of the bath; dry transfer: an additional layer of PDMS is fixed on PMMA to handle PMMA/1L without the stabilizing interface of water. (E) PMMA is removed by acetone leaving the CVD 1L on new substrate.

The means of choice for large flake sizes, as can be produced by CVD, is a polymethylmethacrylate (PMMA)-supported etch transfer method (Fig. 16).^106^ Therefore, PMMA is dropped on a large 1L, spin casted and dried to stabilize the intact lattice (Fig. 16B). In the case of CVDgrown graphene the underlying substrate is a Cu foil, which is *e.g.* etched by a Fe(NO_3_)_3_ solution and washed with water. For a Si/SiO_2_ substrate the removal of the substrate can be achieved by intercalation of water between the hydrophobic polymer/flake and hydrophilic substrate (named wedging transfer method; Fig. 16C). In both cases a subsequent wet-transfer may be performed by decreasing the water level. However, also a dry transfer is possible (Fig. 16D).^107^ Finally, PMMA is dissolved in acetone, rinsed and dried, leaving an intact lattice on the desired substrate (Fig. 16E).

Further methods are reported,^105,108–111^ which are specific for materials, such as oxo-functionalized graphene,^112^ TMDCs^113^ or requirements due to the shape of the substrate.^114^

## 4 Directions of research of intercalated few-layer 2D materials

With thinning down the *z*-direction of layered 2DMs, properties changes, as introduced in the introduction. In this section, we highlight some research directions reported for intercalated few-layered 2DMs. In particular, we will focus on superconductivity, band-gap tuning, magnetic properties, optical properties, energy storage and chemical reactions.

### 4.1 Superconductivity

**Fig. 17:**
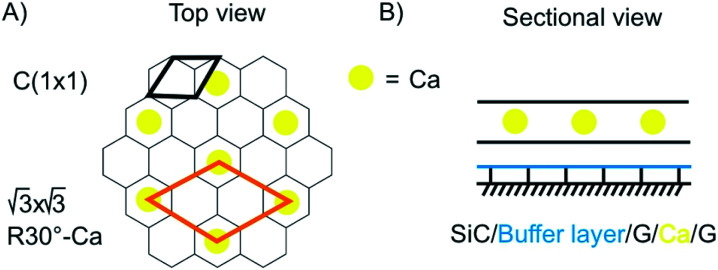
Illustration of the structure of superconductive Ca-intercalated 2L graphene. (A) Top view with graphene lattice in grey and position of Ca atoms in yellow. (B) Sectional view illustrating the layered structure.^124^

Superconductivity is a general research direction to find materials, which transport charge carriers at room temperature without resistance. The search for new superconductive materials is ongoing. In 1987 the Nobel prize in Physics was awarded to J. Georg Bednorz and K. Alex Müller “for their important break-through in the discovery of superconductivity in ceramic materials”.^115^ Thereby, the research is based on the preparation of oxo-cuprates. Currently, Hg_12_Tl_3_Ba_30_Ca_30_Cu_45_O_127_ holds the world record for superconductivity at ambient pressure at 138 K.^116^ Superhydrides, which possess structures of solid atomic metallic hydrogen, such as LaH_10_ (ref. 117) show a transition temperature of astonishing 259 K, however, at 170 GPa.^118^ The current world record holding superconductive materials are brittle and making km-long wires is not in reach. Thus, superconductivity in few-layered 2DMs would be the next superlative. Superconductivity is well-known for carbon materials, such as fullerenes,^119^ carbon nanotubes^120^ and diamond.^121^ For graphene and doped graphene, superconductivity was predicted,^121^ and for bulk CaC_6_, synthesized from graphite and Li–Ca alloy, superconductivity was reported below 11.5 K.^122^ A surprising discovery in this field was reported in 2018 for twisted 2L graphene with a twisting angle of 1.1°. At this “magic-angle” superconductivity is occurring at 1.7 K.^97,123^ In 2016 Ca intercalated 2L graphene (C_6_CaC_6_) was reported to be superconductive at 4 K.^124^ The structure was prepared from epitaxial 2L graphene. First Li atoms were intercalated and then exchanged by Ca (Fig. 17). Although this temperature is lower compared to the bulk, the experiment demonstrates that superconductive properties remain in 2L. Also other intercalated 2DMs are predicted to be superconductive, such as 2L borophene (B_2_MgB_2_) below 23 K.^125^

### 4.2 Band gap tuning

In this section, we highlight the possibilities to tune the electronic properties of 2L and 3L graphene by intercalation. Since graphene lacks a band gap, conventional transistors with *I*_on_/*I*_off_ ratios >10^6^ are by far not possible.^12,126^ Thus, opening a band gap is necessary to generate a transistor behaviour with reasonable *I*_on_/*I*_off_ ratios. Breaking the inversion symmetry in 2L graphene is one way to induce a band gap.^127^ It was reported that dual gating of 2L graphene can open a band gap, reaching even an insulating state.^128^ With tuning the voltages of the top and bottom gate, tuning of the band gap of 2L graphene was demonstrated with values up to 250 eV.^129^ Instead of dual-gating also intercalation/surface chemistry can induce a band gap.

**Fig. 18:**
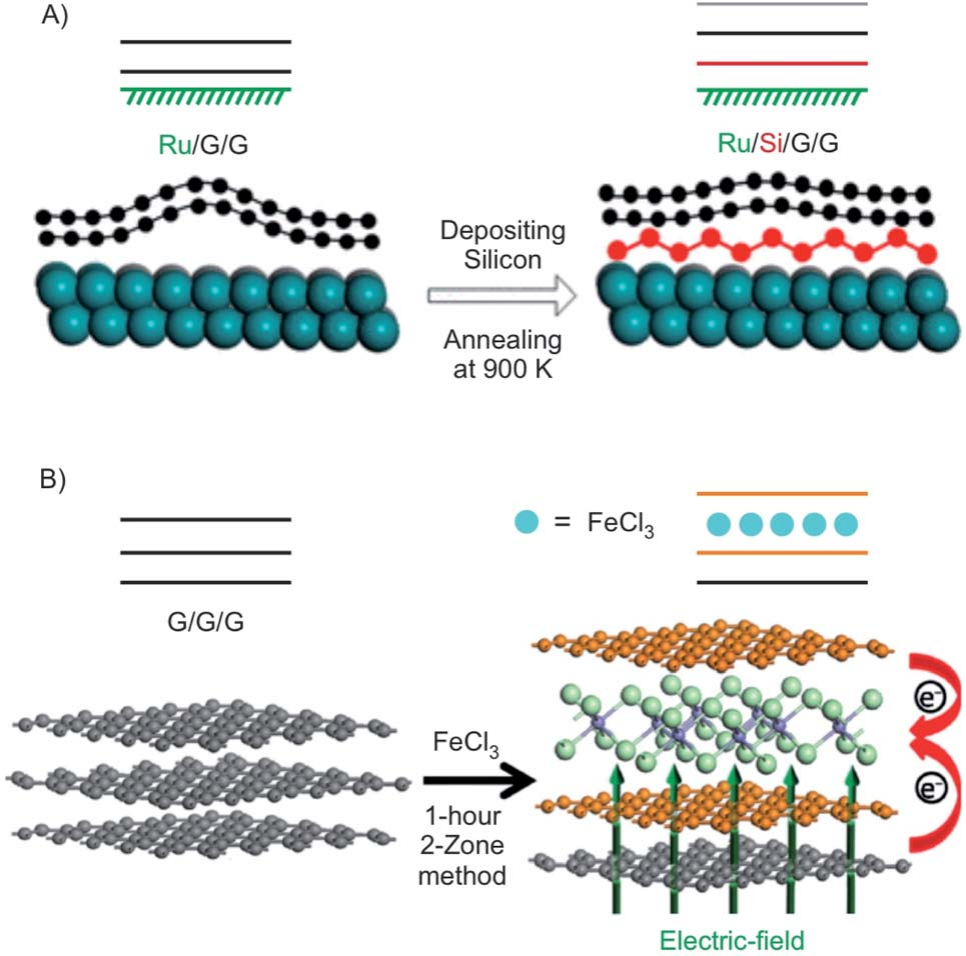
(A) Illustration of the formation of the structure of Ru/Si/G/G with silicene intercalated between Ru and 2L graphene in which silicene induces a band gap. Adapted from ref. 130 with permission from the American Chemical Society, Copyright 2020. (B) Illustration of the intercalation of 3L graphene by FeCl_3_. Reproduced with from ref. 131 with permission from Wiley-VCH Verlag GmbH & Co, Copyright 2020.

Using density functional theory with vdW density functional, it was predicted that 2L graphene can be intercalated between K on the bottom and FeCl_3_ on top to open a band gap in 2L graphene with application relevant 0.4 eV coming into reach.^132^ Experimentally, a 2L of graphene was grown on Ru and subsequently intercalated by silicene (Fig. 18A). It is reported that the layer of silicene, which is intercalated between Ru and the 2L of graphene induces a band gap of about 0.2 eV.^130^ In another approach 3L graphene, prepared by mechanical cleavage, was intercalated by FeCl_3_ by the two-zone method (Fig. 18B). Under the experimental conditions a stage 2 intercalation product is obtained, as evidenced by a splited G peak in the Raman spectrum. A band gap of 0.13 eV was calculated for the experimentally obtained structure. In addition, the authors report that the intercalation compound is stable in ambient conditions.^131^ In general, the decomposition of FeCl_3_ intercalated graphite is kinetically hindered, since water must diffuse into the interlayers. It is described that [FeCl_2_(OH_2_)_4_]^+^ and 4 Cl^−^ are initially formed by the reaction of 4 H_2_O and [FeCl_6_]^3−^. Subsequently, [FeCl_4_]^−^ ions are formed, which are less densely packed.^133^

### 4.3 Magnetism

**Fig. 19:**
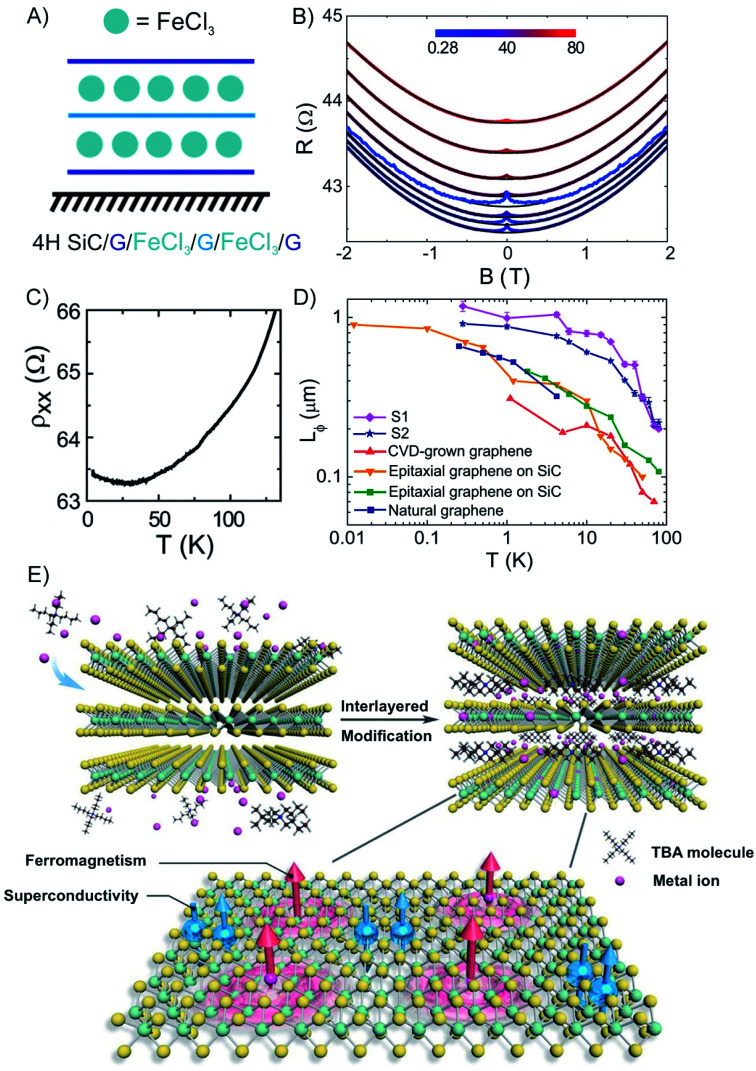
(A) Scheme of FeCl_3_ intercalated 3L graphene. (B) The temperature dependence of the longitudinal resistivity. (C) Magneto resistance at various temperatures (see color-coded legend). (D) The temperature dependence of *Lϕ* for pristine graphene prepared by different methods. The values are compared to other works (original works are referenced in the cited publication) to the estimated values of FeCl_3_-3L graphene. Adapted from ref. 57 with permission from the American Chemical Society, Copyright 2014. (E) Illustration of the interlayer-space-confined chemical design toward the synthesis of TaS_2_ inorganic/organic molecular superlattice with the superconducting regions and ferromagnetic regions in single atomic layers. Reproduced from ref. 141 with permission from Wiley-VCH Verlag GmbH & Co, Copyright 2020.

Among many important properties of 2DMs, the active magnetic response or magnetism has been studied for the development of various applications, including spill oil recovery, targeted drug delivery, and antibacterial interfaces.^134–137^ Few-layered hosts with magnetic intercalants are unique systems to study magnetism in the 2D limit of materials.^47,57,138^ FeCl_3_ has been intercalated into macroscopic scale (1 cm^2^) epitaxial 3L graphene grown on 4H–SiC to a stage 1 compound (Fig. 19A).^57^ The measured magnetoconductance shows a strong weak localization (WL) feature at cryogenic temperatures (<25 K, Fig. 19C). We note that similar WL in resistance have been reported in bulk magnetic-acceptor GICs (FeCl_3_, CoCl_2_),^139,140^ where the abrupt change of resistance has been connected with its magnetic transition.^47^

As shown in Fig. 19B, a detailed study of the temperature dependence of the longitudinal magnetoresistance shows that the WL peak is heavily suppressed when the temperature increases. These findings demonstrate that the intercalation of FeCl_3_ originates from single layer-like hole gases in the stacking with a phase coherence length (*Lϕ*) as large as 1.17 ± 0.08 μm at 280 mK. The temperature dependence of *Lϕ* shows a steep decrease for temperatures higher than ∼30 K (compatible with the 2D magnetic correlations in the plane of FeCl_3_), while *Lϕ* tends to saturate at temperatures lower than ∼4 K (compatible with 3D antiferromagnetic coupling between planes of FeCl_3_).^57^ For temperatures higher than 30 K, a sharper decrease of *Lϕ* is observed in FeCl_3_-FLG, as compared to pristine graphene indicating that randomly oriented magnetic moments in the intercalated FLG are driving excessive dephasing (Fig. 19D). Ferromagnetism and superconductivity are two antagonistic phenomena since ferromagnetic exchange fields tend to destroy singlet Cooper pairs.

The coordination of these two competing phases has been achieved by alternative stacking of superconductor and ferromagnetic layers in vertical heterostructures.^142,143^ However, an interlayer-space-confined chemical design (ICCD) is reported to integrate these two phases in one single-atom-doped TaS_2_ layer, whereby ferromagnetism is observed in the superconducting TaS_2_ layers.^141^ The intercalation of bulky 2H–TaS_2_ crystal with tetrabutylammonium chloride molecules expands its gap between layers, now suitable for single-atom doping *via* co-intercalated Co ions, resulting in the formation of quasi-1L Co-doped TaS_2_ superlattices (Fig. 19E). Furthermore, Co-doped TaS_2_ is exfoliated into ultrathin flakes (20 nm) under ultrasonication and dispersed in DMF, by which their magnetic properties were investigated. Isolated Co atoms are decorated in the basal plane of the TaS_2_*via* replacing the Ta atom or anchoring at a hollow site, wherein the orbital-selected p–d hybridization between Co and adjacent Ta and S atoms induces local magnetic moments with strong ferromagnetic coupling. This ICCD approach can be applied to intercalate various metal ions, enabling the synthesis of a series of crystal-size TaS_2_ molecular superlattices.

### 4.4 Optical properties

#### 4.4.1 Transparency

The combination of transparency and electrical conductivity of materials is the basis for touchscreen applications or organic light emitting devices. Most touch panels use indium tin oxide and the surface resistance is between 5-100 Ω sq^−1^. Thereby, the transmittance at 550 nm is about 85%. Reaching such values using 2DMs as single or multilayer is difficult. Graphene, as thinnest material absorbs 2.3% of light per layer and after chemical doping by *e.g.* TFSA ((CF_3_SO_2_)_2_NH) the resistance dropped to 129 Ω sq^−1^.^144^ With stacking four layers of graphene and doping by AuCl_3_ the sheet resistance can be reduced to 34 Ω sq^−1^.^145^ However, intercalation of few-layer graphene, such as 4L or 5L was demonstrated to reduce the sheet resistance keeping the transparency high.^58^ Thus, exfoliated few-layers, such as 5L graphene, were intercalated by FeCl_3_ at 360 °C using the two-zone method. The realized sheet resistance for intercalated 5L graphene was determined to 8.8 Ω sq^−1^ at an optical transmittance of about 84%.^58^ Large area graphene films (11 × 11 cm^2^) were fabricated by artificial layer-by-layer transfer of AuCl_3_ doped graphene. The sheet resistance of layer-by-layer AuCl_3_ doped 4L graphene was 54 Ω sq^−1^ at 85% transmittance. This method offers not only an improvement of sheet resistance and uniformity but also better environmental stability compared to topmost layer doping.^146^

Metallization of graphite and few-layer graphene, respectively, is possible by intercalation with Li, K or Cs as intercalant.^38,49^ With intercalation the Fermi level is shifted to higher energy. Accordingly, interband optical transitions are suppressed, which can be explained by Pauli blocking. Thus, the transmittance is increased and reaches the visible at high charge-carrier densities.^147^ Since those optical transitions are no longer possible, as those states are filled by the heavy n-doping.

**Fig. 20:**
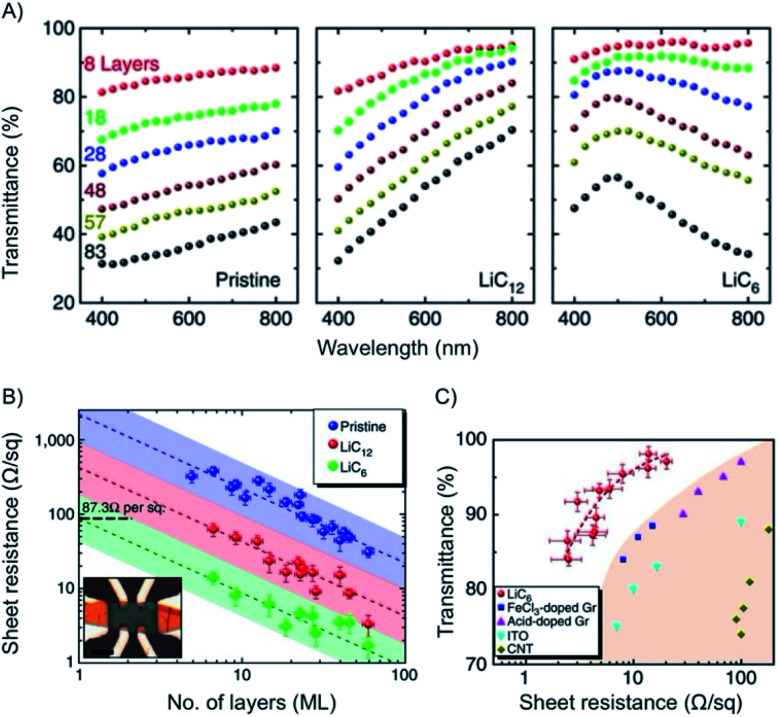
(A) Plots of transmittance *vs.* wavelength for few- and multilayers of graphene and intercalated materials. (B) Plot of the sheet resistance *vs.* number of layers of intercalated materials. (C) Plot of transmittance *vs.* sheet resistance for intercalated materials and other reference materials. Reprinted from ref. 148 with permission from Macmillan Publishers Ltd: Nature Communications, Copyright 2014.

With n-doping of graphene the charge carrier density increases to about 6 × 10^14^ cm^−2^ and therefore the conductivity. The Fermi energy increases to about 1.5 eV.^149^ This concept was studied *in situ* for few- and multilayer graphene.^148^ As depicted in Fig. 20, a maximum in transmittance is reached at 500 nm for multi-layers of graphene, such as 19L with transmission of 91.7%. The results of *e.g.* few-layer graphene (8L) are also shown in Fig. 20.

#### 4.4.2 Optoelectronic properties

**Fig. 21:**
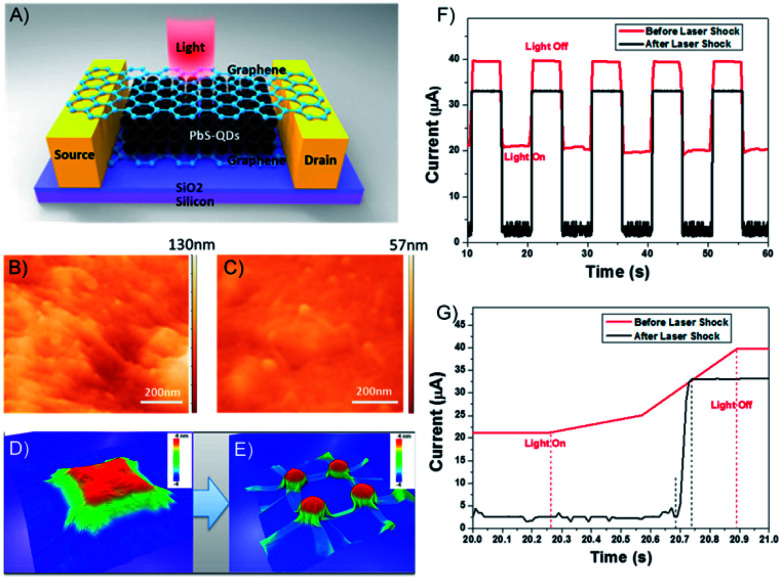
(A) Structure of graphene/PbS/graphene hybrid structures. The channel length between source and drain is 50 μm. AFM image of graphene wrapped on PbS QDs (B) before and (C) after laser shock fabrication. Molecular dynamics simulation results demonstrate graphene wrapping on 3D feature surface (D) before and (E) after the laser shock process. (F) *I vs. t* curve of graphene/PbS/graphene hybrid photosensor before and after the laser shock process at the gate voltage of 20 V and (G) magnified image of (F). Adapted from ref. 158 with permission from the American Chemical Society, Copyright 2017.

Few-layered 2DMs and their heterostructures have shown great promise for new optoelectronic applications,^150,151^ such as high-speed optical communications^152,153^ and wide-optical-bandwidth photodetectors.^154,155^ Graphene has attracted intense research due to its atomic layer thickness, ultrahigh strength and free carrier mobility. Nevertheless, the weak light absorption and zero bandgap of graphene largely limited its application in the optoelectronic field. Thus, graphene-based intercalated structures have been proposed to improve the low optical absorption and quantum efficiency of graphene. Fano-resonant Au plasmonic clusters have been sandwiched between 2L graphene producing a photodetector, where two graphene layers perform as two carrier channels. The photocurrent of the device was enhanced 8 times, which is owing to the hot electrons transfer generated in antenna structure and direct plasmon-enhanced excitation of intrinsic graphene electrons. The internal quantum efficiency for the device achieved up to 20% in the visible and near-infrared regions of the spectrum.^156^ As a low-cost and easy-accessible alternative, rhodamine 6G (R6G) dye with only 1L thickness (0.85 nm) was deposited by a simple dip-coating method to build a graphene–dye–graphene (G–R–G) sandwich photodetector.^157^ The strong π–π interaction force in the G–R–G structure reduced the intermolecular distance, which accelerated the photoexcited charge transfer from the top and bottom graphene to the R6G 1L. The photocurrent and responsivity of the G–R–G device was found to be ∼40 times better than R6G-attached single-graphene device.^157^ However, traditional transfer methods restrict the contact between the top layer of graphene and the underlying intercalant (especially for 0D and 3D intercalant) to grid-space contact, resulting in a weaker transmission in the structure and inevitable artificial scattering. To solve the problem, a graphene/PbS-quantum dots (PbS-QDs)/graphene sandwich structure with seamless 2D/0D contact was fabricated by laser shock imprinting, which opto-mechanically tunes the morphology of 2DMs to perfectly wrap on 0D materials and efficiently collect carriers from the PbS-QDs (Fig. 21A–E). This seamless integrated 2D/0D/2D structure significantly enhanced the carrier transmission, double increase of photoresponse, 20-fold response time and 13-fold photoresponse speed (Fig. 21F and G).^158^

The valley degree of freedom in 2D-crystals recently emerged as a novel information carrier in addition to spin and charge applications.^159^ TMDC-1Ls feature a coupled spin-valley degree of freedom and have emerged as an exciting spin/valleytronic platform. However, the intralayer exciton spin/valley lifetime in 1L TMDCs is limited to tens of nanoseconds due to the unique spin-valley locking behaviour. Achieving long-lived population and polarization lifetimes in TMDC materials is of central importance for their optoelectronic, photonic, and spin/valleytronic applications.

Type II heterostructures, such as WSe_2_/MoSe_2_ have been fabricated to reach long valley polarization times, but precise control of the exciton transformation process (including intralayer to interlayer exciton transition and recombination) and a valley polarization process *via* structural tuning is more challenging. An intermediate layer of hBN was transferred between a WSe_2_/WS_2_ heterostructure. The increased spatial separation with hBN intercalation weakens the electron–hole Coulomb interaction and significantly prolongs the interlayer exciton population and valley relaxation lifetime in vdW heterostructures.^160^ Therefore, WSe_2_/WS_2_ heterostructures with 1L hBN intercalation exhibit a hole valley polarization lifetime of ∼60 ps at room temperature, which is approximately threefold and 3 orders of magnitude longer than that in WSe_2_/WS_2_ hetero-2L without hBN and WSe_2_-1L, respectively.^160^

### 4.5 Energy storage

**Fig. 22:**
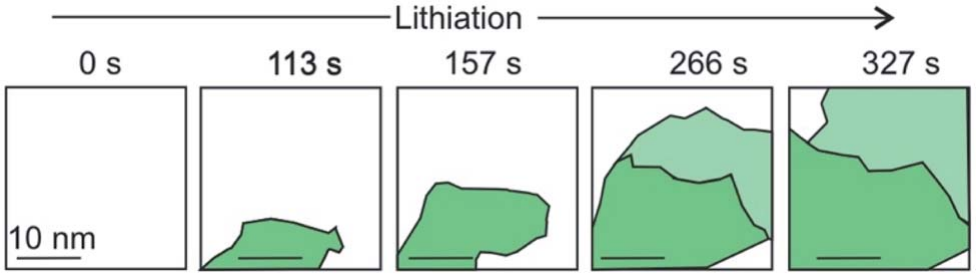
A schematic and simplified illustration of the temporal evolution of lithium intercalation into 2L graphene as evaluated by transmission electron microscopy. Crystal grains are color-coded by their in-plane orientation in green and light green. More details can be found in the original figure.^15^

The intercalation of Li ions into the galleries of a graphite anode is the key step of electrical energy storage in Li-ion batteries that are the major energy storage technology from electric cars to mobile devices. The ideal energy storage device must combine a high energy and power density with a long cycling lifetime. The energy density depends on the amount of charge that can be stored in the material. The power density depends on how fast the intercalant can diffuse into and out of the layered material. These two factors should be combined with a high cycling life time, that is a large number of intercalation/deintercalation cycles without significant performance loss. For the further development of this key technology a thorough understanding of the processes at the atomic level is highly desired. Studying the intercalation at the 2D level allows to follow the electrochemical intercalation process by various techniques *in situ* such as XRD,^161^ Hall measurements^22,93^ and also to follow the process by optical microscopy^89,162^ and even transmission electron microscopy at atomic resolution.^15,93,163^ TEM imaging not only allows to optically follow the intercalation and deintercalation of the ions into the few-layered 2DM, but also identifies areas of varying crystallinity and grain sizes (Fig. 22). As highlighted in chapter 1, Kühne *et al.* have demonstrated the Li intercalation of 2L and 3L graphene deviating from the expected C_6_LiC_6_ compound structure using TEM imaging at atomic resolution. The increased Li storage capacity by super dense ordering of Li in the vdW gap of 2L graphene compared to its bulk counterpart is attributed to the fact that the 2L spread more easily when they are isolated from their bulk crystal. This finding indicates distinct Li storage arrangements with larger storage capacities in 2L compounds compared to their bulk counterparts.

**Fig. 23:**
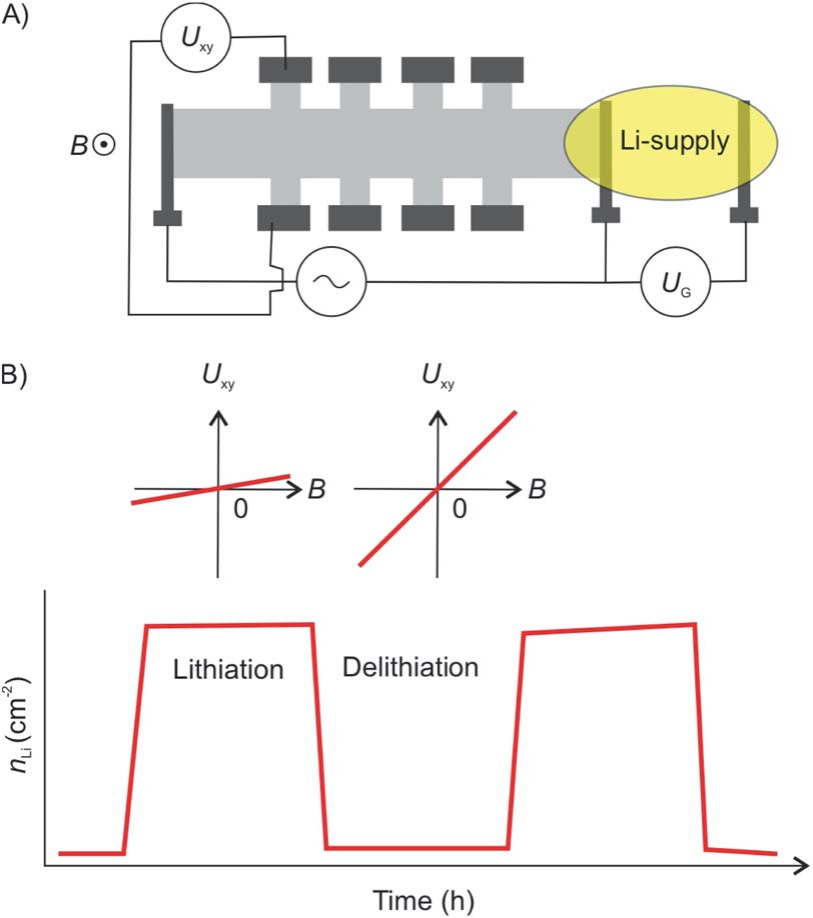
(A) Illustration the 2L graphene device for measurement of Li-ion diffusion rates. The 2L device was shaped into a Hall bar configuration. (B) Lithiation/delithiation cycles visualized by charge carrier concentration extracted from Hall measurements showing full reversibility of the intercalation cycles.^93^

The diffusion of the intercalant into the layered material is an important parameter as it defines the speed at which a device can be charged and discharged. The diffusion rate of Li ions into graphite is relatively low at 10^−8^ cm^2^ s^−1^ resulting in a low power density of Li-ion batteries. By thinning down the electrode to 2L of graphene an exceptional diffusion rate of up to 7 × 10^−5^ cm^2^ s^−1^ was reported.^93^ The diffusion rate was determined by measuring the temporal evolution of the Li density at discrete positions in the device using Hall measurements (Fig. 23A and B). The increased diffusion rate is attributed to the widening of the vdW gap by the intercalated Li ions. Again, this effect can be attributed to the fact that the isolated layers may spread more easily compared to their bulk counterparts.

As a general scheme, tuning the interlayer distance in 2L materials can be used to engineer the energy storage properties. This can be achieved by stacking different 2DMs, creating vdW heterostructures. The intercalation of heterostructures of 2DMs with dissimilar properties may be a promising way to develop high-performance energy storage devices with increasing complexity. The combination of 2DMs allows to make use of the materials advantages by eliminating some of their shortcomings by creating new heterointerfaces and combining the materials electronic properties. For example, the expansion of an electrode upon intercalation is a crucial factor for the cycling lifetime of a battery device. A heterostructure of multi-layered VOPO_4_ with multilayer graphene showed negligible expansion upon electrochemical intercalation and deintercalation with Na^+^, K^+^, Zn^2+^, Al^3+^ cations that was attributed to the in-plane lattice mismatch and the resulting compressive strain on the VOPO_4_ sheets.^164^ As the number of 2DMs is still increasing they are covering a wide range of electronic properties from metallic materials such as Ti_3_C_2_, zero-band gap as in the case of graphene, semiconducting such as MoS_2_ or black phosphorous to large band gap materials such as hBN. The combination of these materials creates a large library of new materials that allow to tune their electrode properties in energy storage devices.^165^

**Fig. 24:**
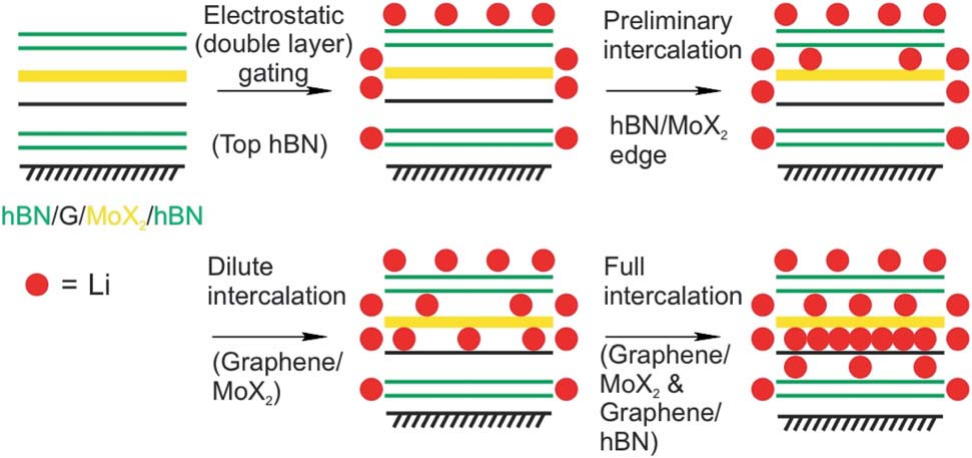
Proposed four-step Li intercalation mechanism for a hBN (green)/MoX_2_ (yellow)/graphene (black)/hBN heterostructure, evidenced by *in situ* HRTEM imaging.^87^

Mechanical strain induced by the lattice mismatch in vdW heterostructures has been demonstrated in multi-layered materials to control various materials properties.^166^ Mechanical strain induced by the lattice mismatch of vertically stacked multi-layered MoS_2_ and thin carbon layers was utilized to influence the pathways of electrochemical reactions upon Li intercalation. By engineering the strain in the system the chemical reaction could be influenced from intercalation in the pristine MoS_2_ system to the chemical conversion to Lithium sulfide in the case of thin carbon layers stacked MoS_2_ multilayers.^86^ On the 2L level, the Li intercalation in vdW heterostructures of graphene and molybdenum dichalcogenide (MoX_2_, X = S, Se) encapsulated in hBN showed a 10-fold increase of accumulated charge compared to MoS_2_/MoS_2_ 2L devices as demonstrated by Bediako *et al.*^87^ They fabricated five vdW heterostructures of graphene, TMDCs and hBN in which graphene and MoX_2_ layers are encapsulated by hBN and employed as the working electrode of an electrochemical cell. By using the Hall potentiometry method they followed the Li intercalation upon sweeping the potential in various heterostructures. They elucidated the mechanisms of the Li intercalation at the various heterointerfaces. By combining the results of transmission electron microscopy, *in situ* magnetoresistance, optical spectroscopy techniques, *ab initio* calculations and low-temperature quantum magneto-oscillation measurements they derived an intercalation mechanism that describes the intercalation into a graphene/MoX_2_ 2L encapsulated by hBN (Fig. 24). The proposed mechanism showcases the varying intercalation processes at the heterointerfaces of the 2DMs that can be understood by the Li binding energies calculated for the various heterointerfaces. Their study revealed that the capacity of the graphene/MoX_2_ heterointerface is more than 10-times larger than the capacity of the other heterointerfaces (graphene/hBN, MoX_2_/hBN). Furthermore, they observed a decreased onset intercalation voltage of graphene/MoX_2_ that is about 0.5 V larger than that of a graphene/hBN heterostructure shown in a previous study.^22^ MoS_2_ undergoes a phase transition from the initial semiconducting H-phase to the metallic T'-MoS_2_ phase after charge transfer from Li. This phase transition lowers the activation barrier for further Li intercalation.

### 4.6 Chemical reactions

The process of chemical reactions takes basically place on the surface of a bulk material. By thinning down the material, properties change such as the reactivity or absorption and emission properties for example in QDs, as outlined below. Not only by increasing the surface, but also by intercalation, layers become more accessible to the reactant or are activated by the intercalant. In principle, chemical reactions can be accelerated or observed for the first time in intercalated structures.

**Fig. 25:**
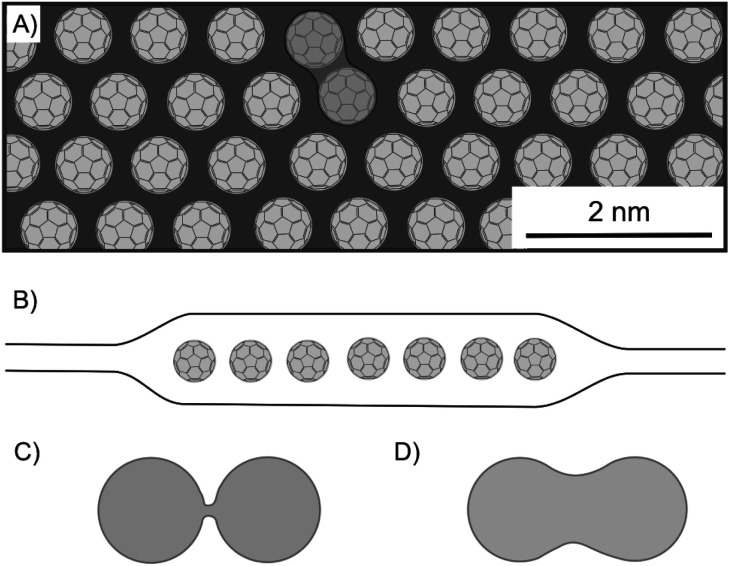
Fullerenes encapsulated between graphene layers and investigated by STEM analysis. (A) Illustration of a 1L of hexagonally ordered fullerenes, showing a bonded couple. (B) Schematic illustration of the heterostructure of graphene/C_60_/graphene. (C) Illustration of a loosely bound fullerene dimer and (D) of a fullerene peanut dimer.^101^

One of the first heterostructures with mixed dimensional materials (2D/0D/2D) consists of a graphene-coated TEM grid with thermally evaporated fullerenes and transferred graphene as top-layer (Fig. 25A and B).^101^ Due to the low fullerene moving barrier of 5 meV, the self-assembly of the 0D component leads to big areas of single-layered, hexagonally closed packaging in between graphene. It turned out that the lattice spacing is 4–5% smaller than in equivalent 3D crystallites, while the fullerenes remain rotationally active. Although graphene acts as a protecting layer during STEM, the e-beam irradiation activates the molecules and causes bond formation between fullerenes forming a peanut-like dimer, as shown in Fig. 25A and D, a process which is supported by calculations starting from a loosely bound dimer of fullerenes (Fig. 25C). Due to the regular structure of the graphene lattices, those lattices can be filtered out in TEM images, enabling the unhindered observation and study of the intercalant. The encapsulation within graphene can also be applied to other 2DM, as demonstrated for MoS_2_.^167^ With this approach, molecular structures and their dynamics can be analyzed.

**Fig. 26:**
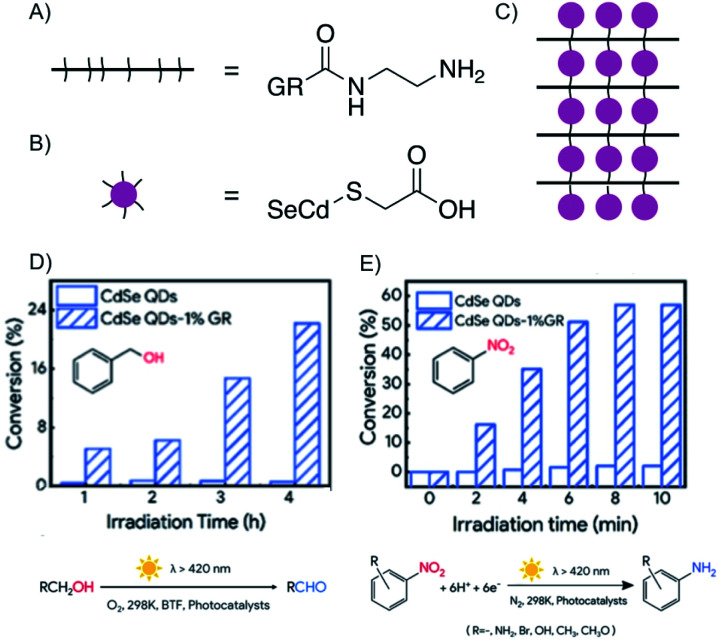
Enhanced electron transfer in covalent bonded CdSe QDs/reduced graphene oxide (GR) heterostructure obtaining enhanced photoredox conversion. (A) Schematic and chemical structure of ethylenediamine functionalized graphene. (B) Schematic and chemical structure of sulfanylacetic acid functionalized CdSe QDs. (C) Schematic illustration of CdSe QDs/reduced graphene oxide heterostructure. (D) Comparison of conversion efficiency of the heterostructure and the sole QD for the oxidation of benzyl alcohol to the aldehyde. (E) Comparison of the conversion efficiency of the heterostructure and the sole QD for the reduction of nitrobenzene to aniline. Adapted from ref. 168 with permission from the American Chemical Society, Copyright 2019.

The factor for improving reactivity using the concept of heterostructures is significant for QDs.^168^ QDs gained attention in photocatalysis for their high quantum yields and specific quantum confinement, but lacked in photostability, long lifetime and fast electron transfer. However, Huang *et al.* fabricated a heterostructure that did overcome those drawbacks (Fig. 26C).^168^ The heterostructure is fabricated layer-by-layer, by electrostatic self-assembly in aqueous solution, with positively charged graphene oxide, which was functionalized by ethylenediamine (Fig. 26A), and negatively charged CdSe QDs, modified by sulfanylacetic acid (Fig. 26B) connecting graphene oxide layers and QDs by peptide bonds. Subsequently, reduction generates reduced graphene oxide, which is further termed as graphene. It was shown that graphene improves the photostability, adsorption of reactants and the separation of excitons of QDs due to fast electron transport.

The oxidation of aromatic alcohols to aldehydes (Fig. 26D), such as benzyl alcohol to benzaldehyde, and the reduction of nitroaromatics to amino aromatics, here to aniline (Fig. 26E), are enhanced compared to mere QDs, because the reactivity is ascribed to holes and electrons as well as hydroxyl radicals. The heterostructure in this example possesses enhanced photoredox activity due to the artificial intercalation of QD between graphene.

## 5 Perspectives

We presented the emerging field of research of the intercalation of 2DMs with a special focus on intercalated 2L and 3L systems. As described in the last Section 4.6, a heterostructure assembled of a 2DM and a photosensitizer is promising for developing photocatalysts. However, the self-assembly strategy used in that example does not stop at the thinnest intercalated counterpart to the bulk but leads to 3D structures. Although not investigated yet, intercalated 2L systems bear a larger area for the interaction with substrate molecules and will possibly lead to higher quantum yields and thus, more efficiency. With thinning intercalated 2DMs the ultimate surface access is possible, in particular once intercalated few-layered 2DMs are placed on a membrane support, giving access to both sides. Furthermore, manipulating the intercalant can be highly effective in such systems, since only one layer of a 2DM is shielding the intercalant. Therefore, we speculate that novel concepts for sensing devices will emerge and unforeseen properties will be found, as already described for 1L or 2L graphene and the 2DMs family in general. Another benefit of thinning materials to the limit is the ability to observe elemental processes by spectroscopy probing the complete system compared to the surface as only a minor part of the bulk, *e.g.* by Raman spectroscopy. Moreover, microscopes improve and can monitor intercalation processes on the atomic scale, leading to a better understanding of even application relevant charge storage systems. Assembly techniques are already established to fabricate complex layered systems, which are based on 2DMs as well as molecules, respectively. At the same time, different scientific fields come together and envision novel applications.

In this review, we focused on the fields of superconductivity, band gap tuning, magnetic ordering, optical properties, energy storage and chemical reactions. However, with advancing the field of intercalated few-layered 2DMs more functional systems with a defined focus on specific applications will emerge. Possible highlights in sensing applications can be imagined since it is already a highly active field of research relevant for all kinds of science, *e.g.* for sensing of small environmental molecules, monitoring biologically relevant species in cells, light sensors or mechanical force sensors, to mention only some. Finally, with increasing the understanding of intercalated 2DMs, it will be possible to gain more control over the chemistry, which can be induced close to the surface. Thus, there is a good chance to develop more energy-efficient chemical reactions with even accelerated kinetics and specificity.

## Conflicts of interest

There are no conflicts to declare.

## Supplementary Material
